# APCformer: an aggregation-perception enhanced convolutional transformer network for MI-EEG decoding

**DOI:** 10.3389/fnins.2026.1766883

**Published:** 2026-03-23

**Authors:** Jiangyin Huang, Jiaxiang Zou, Xiner Li, Yanze Cai, Binyang Lin, Yangjie Li, Xiaohuan Xia

**Affiliations:** 1 School of Electrical Engineering and Automation, Xiamen University of Technology, Xiamen, China; 2 Xiamen Key Laboratory of Frontier Electric Power Equipment and Intelligent Control, Xiamen University of Technology, Xiamen, China

**Keywords:** EEG decoding, information aggregation, interactive sharing, motor imagery, multi-scale feature

## Abstract

Electroencephalogram (EEG) decoding is essential for Brain-computer interfaces (BCI) systems to predict brain activity. However, existing methods usually suffer from two core problems: (1) existing networks lack effective interaction mechanisms and insufficiently capture spatial-temporal dynamic features, leading to the loss of critical fine-grained information; (2) the modeling of long-range dependencies and local features is unbalanced, making it difficult to adapt to the temporal characteristics of EEG signals. To address these issues, this paper proposes an Aggregation-Perception Enhanced Convolutional Transformer (APCformer) network. The network adopts a branch-interactive structure as its main body and jointly extracts shallow features via multi-scale spatial-temporal convolution; an Adaptive Feature Recalibration (AFR) module is embedded to realize cross-scale feature interaction and enhancement of critical fine-grained features. The Position-aware Enhancement (PAE) module is utilized to integrate learnable positional encoding, improving the ability of deep networks to characterize the temporal positional relationships of EEG sequences and enhancing adaptability to temporal dynamic features. We further propose a Sparse Information Aggregation Transformer (SAT), which combines the attention mechanism with the maximum attention mechanism to achieve a balanced modeling of global long-term dependencies and local fine-grained features. Experimental results on the public BCI-IV 2a and BCI-IV 2b datasets show that APCformer achieves superior performance in EEG decoding tasks, with average decoding accuracies of 85.53% and 89.15%, respectively. These results highlight APCformer's strong capability in handling complex EEG features and dynamic patterns, effectively improving the efficiency and accuracy of EEG decoding.

## 1 Introduction

Brain–computer interfaces (BCI) is a cutting-edge technology emerging from the intersection of neuroscience, biomedicine, and engineering, enable direct communication between human thoughts and external devices. Their applications are gradually extending into everyday life (Willett et al., 2021; Mansour et al., 2025). With the rapid development of BCI, motor imagery-based electroencephalography (MI-EEG) has been widely utilized in BCI research (Hsu and Cheng, 2023). By decoding the brain's electrical activity, MI-EEG allows individuals to operate various devices using only their thoughts, offering vast possibilities for future lifestyles (Liu et al., 2025). However, EEG signals are inherently weak physiological electrical signals characterized by nonlinearity, non-stationarity, and high dimensionality. They are also highly susceptible to external interference, making accurate decoding a highly challenging task (Guo et al., 2025; Ke et al., 2024).

Accurate EEG signals decoding is a critical foundation for the stable operation of BCI systems. At the application level, BCI technology is closely tied to EEG decoding, with the realization of the former largely depending on the effectiveness of the latter. In EEG decoding, effective feature selection plays a pivotal role. Traditional feature extraction methods primarily focus on the time, frequency, and spatial domains, yet these approaches typically capture only a single, specific aspect of EEG information (Guan et al., 2025). As a result, recent research has explored multi-domain feature fusion methods that combine various types of features to obtain richer EEG representations (Zhang et al., 2021; Chen et al., 2025). Although such approaches can extract discriminative multi-domain features, they often rely on prior knowledge during the extraction process, which may lead to the loss of a substantial amount of valuable information (Geng et al., 2022).

With continuous breakthroughs in EEG decoding technology, which play a decisive role in advancing efficient human–computer interaction, early EEG decoding methods were primarily based on traditional machine learning algorithms (Zhao et al., 2019). For example, (Richhariya and Tanveer 2018) achieved epileptic signal recognition by improving the support vector machine (SVM). (Wei et al. 2021) performed EEG classification using the filter bank common spatial pattern (FBCSP) method. With the enhancement of computational power, deep learning methods have gradually replaced traditional machine learning approaches and become mainstream, among which convolutional neural networks (CNN) are the most widely used for EEG decoding. (Schirrmeister et al. 2017) explored end-to-end EEG decoding using a deep convolutional neural network (DeepConvNet), achieving performance comparable to that of FBCSP. (Lawhern et al. 2018) developed EEGNet, a compact network specifically designed for EEG analysis based on depthwise separable convolution, which achieved high performance across various benchmark tasks. (Salami et al. 2022) proposed an interpretable network (ITNet) derived from CNN that integrates Inception modules and dilated causal convolutions, leading to improved performance on public datasets. However, these CNN-based models tend to overemphasize local features when dealing with the complex and dynamic characteristics of EEG signals. As a result, they struggle to effectively capture long-range dependencies within EEG data, which limits further improvements in EEG decoding accuracy.

In contrast, the Transformer architecture, by virtue of the powerful sequence modeling and long-range dependency capture capabilities of the self-attention mechanism, has provided novel approaches for the global temporal feature modeling of EEG signals, making Transformer-based decoding methods a research focus in the MI-EEG field. To balance the advantages of CNNs in local feature extraction and the global modeling capabilities of Transformers, many studies have begun to attempt combining different model structures with Transformers, aiming to improve overall performance through model complementarity. For example, (Song et al. 2022) proposed the EEG Conformer model, which extracts local spatio-temporal features using convolution while employing a self-attention mechanism to capture global temporal dependencies, achieving accurate EEG signal decoding. (Gong et al. 2023) designed the Attention-based Convolutional Transformer Neural Network (ACTNN), which demonstrated excellent performance and cross-subject robustness on the SEED emotion dataset. Zhao W. et al. (2024) employed a convolutional module similar to EEGNet to extract local features and used a multi-head attention mechanism to model global dependencies, with their proposed CTNet showing outstanding performance on corresponding datasets. However, these models may overlook fine-grained local features when extracting EEG representations.

In recent years, a number of cutting-edge studies have further expanded the technical boundaries of the Transformer architecture in the field of MI-EEG decoding. Among them, (Liu et al. 2024) proposed an MSVTNet network, which combines multi-scale CNNs and Transformers to extract local spatial-temporal features and cross-scale global coupled features respectively, and uses auxiliary branch loss to optimize the parameter imbalance problem, achieving dual improvement in decoding performance and robustness. (Han et al. 2025) proposed a spatial-spectral and temporal dual-prototype learning framework (SST-DPN), which realizes efficient feature modeling through spatial-spectral fusion and multi-scale variance pooling, and introduces dual-prototype learning for the first time to enhance the intra-class aggregation and inter-class discriminability of features, which can alleviate small-sample overfitting, with high model accuracy, high computational efficiency and no need for preprocessing. (Zhao et al. 2025) proposed a CNNViT-MILF-a dual-branch collaborative architecture, which uses CNNs to extract local spatial-temporal features, models global temporal dependencies through Vision Transformer (ViT), and adopts a ViT-dominated late fusion strategy to integrate local and global features, realizing the synergistic enhancement of CNNs and ViT.

In addition, the academic community has also carried out extensive research focusing on multi-scale feature fusion, collaborative optimization of CNN and Transformer, feature representation enhancement and other related directions, and proposed a series of improved CNN-Transformer architectures for MI-EEG decoding, which also provides an important reference foundation for the research of this paper. (Tao et al. 2023) proposed the ADFCNN model, which combines dual-scale fusion and attention mechanism, enabling it to overcome the limitations of single-scale feature extraction and improving the performance of the model. (Yang and Liu 2024) proposed the MSFCNNet, which enhances feature extraction and the capture of spectral and spatial features through multi-head self-attention mechanism and multi-scale inputs. (Zhao et al. 2025b) proposed MSCFormer and TCANet (Zhao et al., 2025a), both of which are advanced models for MI-EEG classification. Among them, MSCFormer extracts local spatiotemporal features through a multi-branch multi-scale convolutional network and further optimizes the decoding performance of the model by combining Transformer to model the global dependency relationships. TCANet integrates local and global features gradually through multi-scale convolution modules, time-domain convolution modules, and stacked multi-head self-attention, and has been systematically evaluated on public datasets, achieving good results.

Although the aforementioned works have achieved optimization of CNN and Transformer architectures from various aspects and made a series of progress in MI-EEG decoding tasks, further improvements are still needed. First, in terms of multi-scale feature modeling, most methods simply concatenate multi-scale features after parallel extraction, resulting in insufficient multi-scale feature extraction and lack of effective cross-scale feature interaction and adaptive screening mechanisms. For example, MSVTNet, MSCFormer, TCANet and other methods have not designed a dedicated cross-branch interaction mode, and only complete information fusion through feature concatenation, which cannot realize information sharing of features at different scales and enhancement of critical fine-grained features, easily leading to the loss of effective information with low discriminability. Second, due to the strong temporal dependence of EEG signals, in the process of learning deep features in most decoding models, fine-grained information along spatial and temporal dimensions is often ignored, making it impossible to ensure the temporal integrity of deep fine-grained features. For instance, ADFCNN, MSFCNNet and other methods only adopt the standard Transformer encoder structure, using schemes of superimposing fixed positional encoding on the input layer or no positional information; in deep networks, positional information gradually degrades with convolution operations, affecting the model's temporal modeling of EEG signals. Third, most methods cannot focus on local high-value features while modeling long-range dependencies, resulting in an imbalance between global long-range dependency modeling and local fine-grained feature modeling. For example, methods such as CNNViT-MILF-a, MSCFormer, and TCANet all rely on the global self-attention mechanism of Transformers, which has high computational complexity; while improving the global feature modeling capability, it weakens the capture of local fine-grained features, making it difficult to achieve dual optimization of decoding accuracy and computational efficiency.

To address the limitations discussed above, we proposes a multi-scale interactive APCformer network for EEG signal decoding. The research focuses on the optimization and improvement of the CNN-Transformer hybrid architecture, enhancing the network's ability to capture local and global fine-grained features and its cross-scale correlation. The main contributions of this work are as follows:

- To address the issues of the absence of multi-scale feature interaction and the insufficient capture of spatiotemporal dynamic fine-grained features, a multi-scale information interaction sharing network is designed. It breaks through the limitation of fixed receptive fields in convolution and incorporates an AFR module. Through cross-branch interaction, it enhances the model's perception and learning ability of key fine-grained features, achieving the complementation and selection of multi-scale features.
- To address the issue of poor temporal dynamic adaptability of EEG signals in deep networks, a PAE module was constructed to focus on the identification of deep fine-grained features, while integrating learnable feature position encoding information to deeply model the temporal correlations of EEG sequences and enhance the model's adaptability to the non-stationary temporal features of EEG.
- To address the imbalance between the overall long-term dependency and the local detail modeling, the SAT module was proposed. The EEG signals were divided into blocks using a sliding window. Through sparse filtering and core representation aggregation strategies, combined with the linkage of aggregation attention and the highest attention mechanism, the balance between the model's decoding accuracy and efficiency was achieved.
- We conducted experiments on the BCI-IV 2a and BCI-IV 2b public datasets. Compared with the mainstream methods in the field, APCformer demonstrated superior learning ability and performance.

The rest of this article is organized as follows. Section 2 provides a detailed description of the network architecture. Section 3 introduces the experimental design. Section 4 focuses on comparative experiments and analyses. Section 5 discusses this article and looks forward to the future. Finally, Section 6 summarize this article.

## 2 Methods

### 2.1 Overview of APCformer

**Figure 1:**
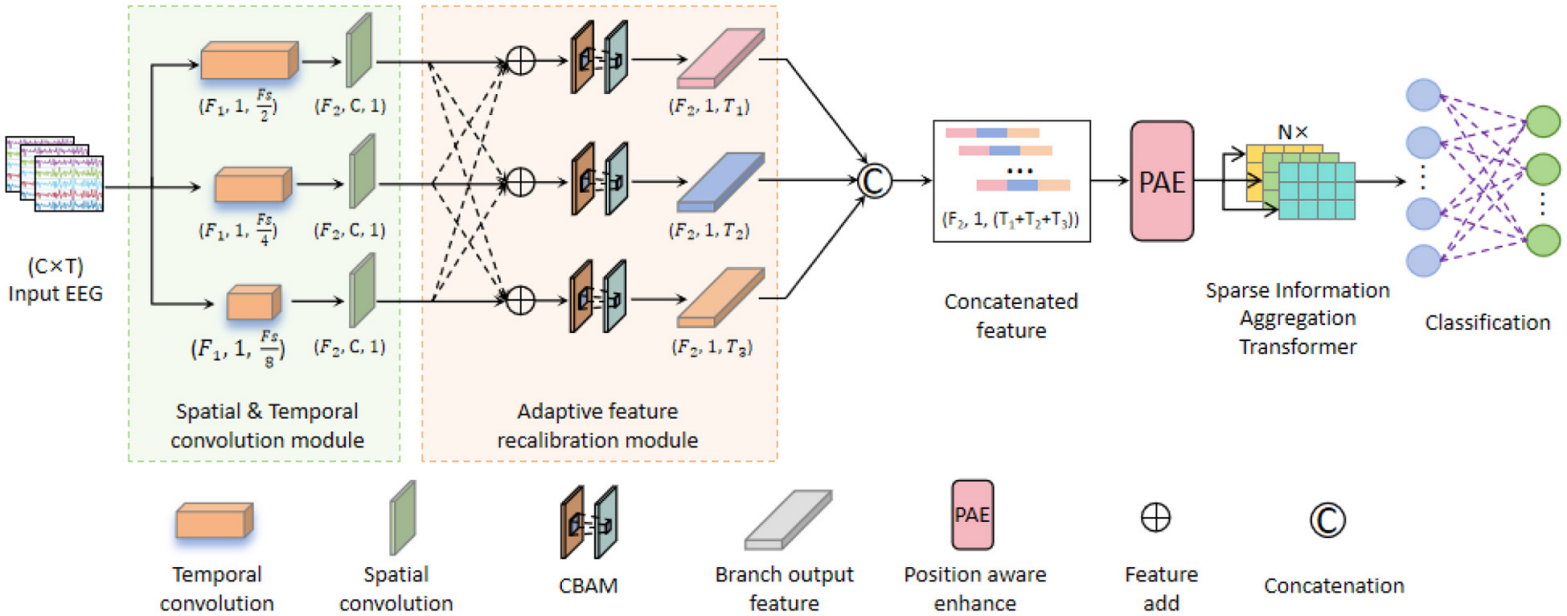
Overall architecture of the APCformer network, which consists of five main components: STConv module, AFR module, PAE module, SAT module, and the classification module. Among them, CBAM is a Convolutional Block Attention Module.

In order to effectively decode the spatiotemporal dynamic information of EEG signals, we propose APCformer, a novel EEG decoding network, whose overall architecture is illustrated in Figure 1. The network comprises five main components: Spatio-temporal convolution module (STConv), AFR, PAE, SAT, and classification module. Batch EEG data are first fed into the STConv to extract multi-scale shallow local features. The AFR module then highlights key spatiotemporal features, which are subsequently passed into the PAE module to extract deep fine-grained features and perform adaptive feature encoding. These enhanced features are further refined by the SAT module to extract long-range dependencies and local associations, achieving effective fusion of local and global representations. Finally, the classification module outputs the decoding result. The remainder of this section provides a detailed explanation of the APCformer architecture and its components. Specific parameters are listed in Table 1.

**Table 1:** Parameters of the APCformer.

**Module**		**Layer**	**Size**	**Output**
Input		-	B = 32	(1, C, T)
STConv	Branch-1	Temporal Conv	(*F*_1_, 1, Fs/2)	(*F*_1_, C, T)
	Branch-2	Temporal Conv	(*F*_1_, 1, Fs/4)	(*F*_1_, C, T)
	Branch-3	Temporal Conv	(*F*_1_, 1, Fs/8)	(*F*_1_, C, T)
	General	Spatial Conv	(*F*_2_, C, 1)	(*F*_2_, 1, T)
		BatchNorm	axis = –1	-
		Activation	ELU	-
		AvgPooling	(1, 75)	(*F*_2_, 1, T/75)
		Dropout	0.3	-
AFR		Add	-	(*F*_2_, 1, T/75)
		CBAM	-	(*F*_2_, 1, T/75)
Fusion		Concatenation	-	(*F*_2_, 1, T/25)
PAE		Enhance Conv	(*F*_2_, 1, 3)	(*F*_2_, 1, T/25)
		Enhance Conv	(*F*_2_, 1, 7)	(*F*_2_, 1, T/25)
		BatchNorm	axis = –1	-
		Add	-	(*F*_2_, 1, T/25)
		Activation	ELU	-
		AvgPooling	(1, 3)	(*F*_2_, 1, T/75)
		Dropout	0.3	-
		Rearrange	-	(T/75, *F*_2_)
		PE	-	(T/75, *F*_2_)
SAT		Attention	-	(T/75, *F*_2_)
Classifier		Flatten	-	(*F*_2_×T/75)
		Dense	Softmax	Class

Among them, *C* = the number of channels, *T* = the number of sampling points, *Fs* = the sampling frequency, *B* = the batch quantity, *Class* = the number of classifications, *F*_1_ and *F*_2_ represent the number of filters corresponding to the convolutional layer, *F*_1_ = 16, *F*_2_ = 32. The specific parameters of SAT can be found in Section 4.4 - Model parameter sensitivity analysis.

### 2.2 Spatio-temporal convolution module

To break through the limitations of single convolutional perception and achieve multi-scale feature processing, the shallow local features of EEG are extracted through multi-scale STConv, enhancing the feature recognition ability without increasing the depth of the network. The first core component of the network is inspired by (Schirrmeister et al. 2017); (Lawhern et al. 2018), with a total of three branches set up. Each branch successively has a temporal convolutional layer, a spatial convolutional layer, a layer normalization, an ELU activation function layer, an average pooling layer, and a Dropout layer. The composition of each branch is roughly the same, all adopting double-layer convolution as the shallow feature encoder. However, there are differences in the design of temporal convolution, with 16 large-kernel convolution of different scales respectively as suggested by Zhao W. et al. (2024), namely (1, *Fs*/2), (1, *Fs*/4) and (1, *Fs*/8), extracting rich temporal features through differentiated receptive fields. Spatial convolution uses 32 (C, 1) convolution kernels to adapt the number of sampling channels. After convolution, batch normalization is added to alleviate the covariate offset problem, and the nonlinear expression ability of the model is enhanced through ELU. Subsequently, the average pooling layer is used to reduce redundant features, and Dropout is employed to reduce the risk of overfitting. Eventually, more representative shallow spatio-temporal features are output.

### 2.3 Adaptive feature recalibration

Cross-scale correlation can effectively enhance the model's ability to perceive multi-scale features, and improve the model's performance in recognizing complex patterns and achieving effective generalization. In multi-branch network structures, the feature scales and temporal locality corresponding to different branches are different, and there are certain internal correlations among the features of each branch. Fully exploring and utilizing these internal correlation information is of great help for subsequent feature extraction (Zhao P. et al., 2024; Cai et al., 2025). The AFR module designed for this purpose can achieve information sharing among branches through interactive feature stacking. Its interaction structure is related to the inherent characteristics of EEG signals. Different time feature scales and temporal locality corresponding to each branch are independently encoded, containing irreplaceable fine-grained discriminative features. If directly merged, the unique feature information of each branch will be blurred and diluted, resulting in the loss of key information with discriminative power in the weak EEG signal from the root (Cai et al., 2025). The interaction structure of the AFR module retains the features of each branch and adds the features of the other branches, allowing each branch to efficiently obtain complementary information from other branches while completely preserving its own unique feature representation. This can effectively avoid the loss of key information and achieve cross-scale association enhancement among branches.

**Figure 2:**
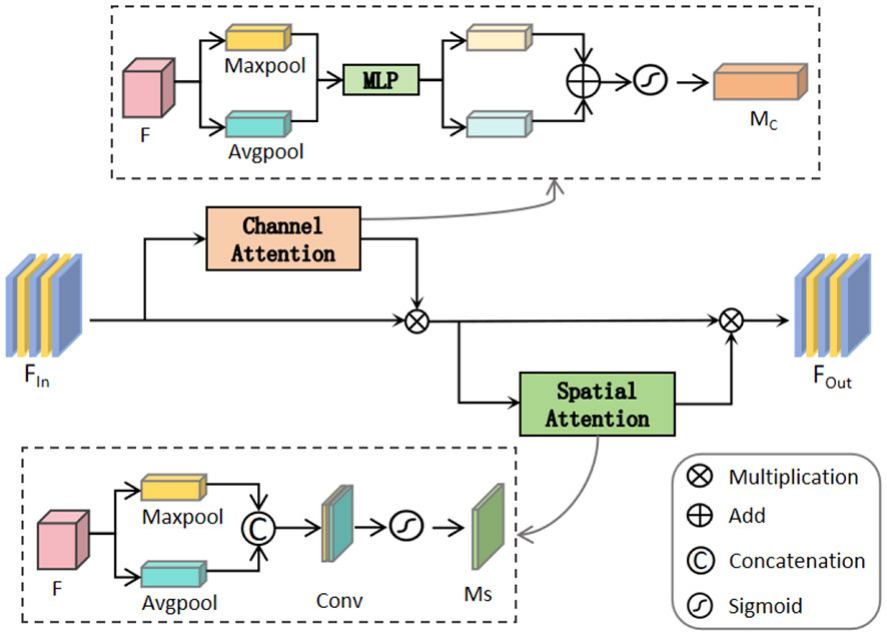
CBAM structure. It is mainly composed of channel attention and spatial attention. Among them, while Conv denotes a 3 × 3 convolution.

Inspired by (Tang et al. 2023), based on the interaction mechanism, introduces the Convolutional Block Attention Module (CBAM) (Woo et al., 2018) to recalibrate the channels and spatial features, enhancing the model's sensitivity to important information. Since EEG signals contain both rhythmical feature differences in different channels and local spatial distribution patterns in the time dimension, CBAM can act on the interaction features that retain branch specificity, and can better adapt to the characteristic requirements of EEG signals. Through the dual-branch collaboration calibration of channels and spaces, CBAM can specifically enhance the weights of key channels within each branch, and further focus on the local time periods with significant discriminative properties in the time series. The structure is illustrated in Figure 2. In CBAM, the channel attention processes the input features through global max pooling downsampling and global average pooling downsampling, and then inputs them into multiple layers of perceptrons (MLPs). Subsequently, these features are added and processed through the Sigmoid activation function to generate channel weights. The spatial attention also processes the input features through global max pooling downsampling and global average pooling downsampling, and then concatenates the two features along the channel dimension. Finally, it generates spatial weights through convolution and the Sigmoid activation function. These weights are applied successively to the channel features and spatial features. Subsequently, the features of each branch are effectively fused and input into the PAE for deep refinement processing.

### 2.4 Position aware enhancement

**Figure 3:**
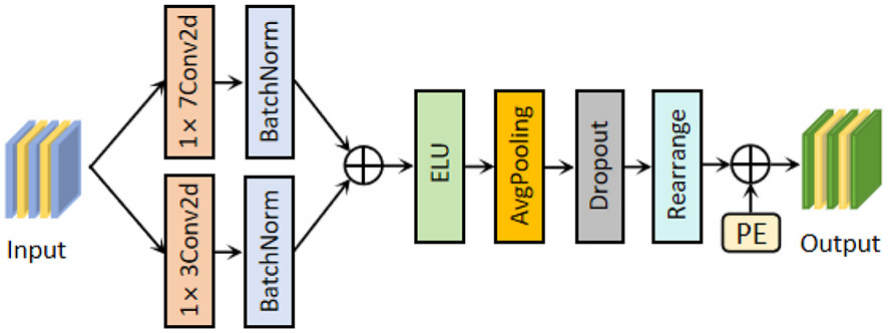
Structure of the PAE module, which features undergo parallel enhanced convolutions, followed by dimensional transformation, and positional encoding information is added to the sequence.

In previous decoding models, fine-grained information along spatial and temporal dimensions was often lost. PAE adopts a parallel small kernel convolution structure and can capture deep and fine-grained local features (Altaheri et al., 2022), as shown in Figure 3. Specifically, PAE further extracts features along the temporal dimension using 32 convolutional kernels of sizes (1, 3) and (1, 7). After batch normalization and feature fusion, the features are activated by the ELU function. An average pooling layer with kernel size (1, 3) and a dropout layer are then applied to optimize the model and address EEG adaptability issues that arise from network deepening. Subsequently, the feature dimensions are linearly transformed, and a learnable positional encoder is introduced. A trainable matrix with the same dimensionality as the input features is randomly initialized, following a standard normal distribution to generate the random tensor. During model training, the parameters of the position encoder will participate in backpropagation to calculate the gradient, continuously updated as the network is trained, and autonomously perceive the position correlation. In the feature fusion stage, the position vectors generated by the position encoder are fused element-by-element with the features after linear transformation, enabling the network to perceive the position information of each feature (Dosovitskiy et al., 2020; Xie et al., 2022). Finally, the features containing location encoding information will be transferred to the SAT to explore complex dependency relationships, further enhancing the recognition ability of APCformer for EEG signals.

### 2.5 Sparse information aggregation transformer

**Figure 4:**
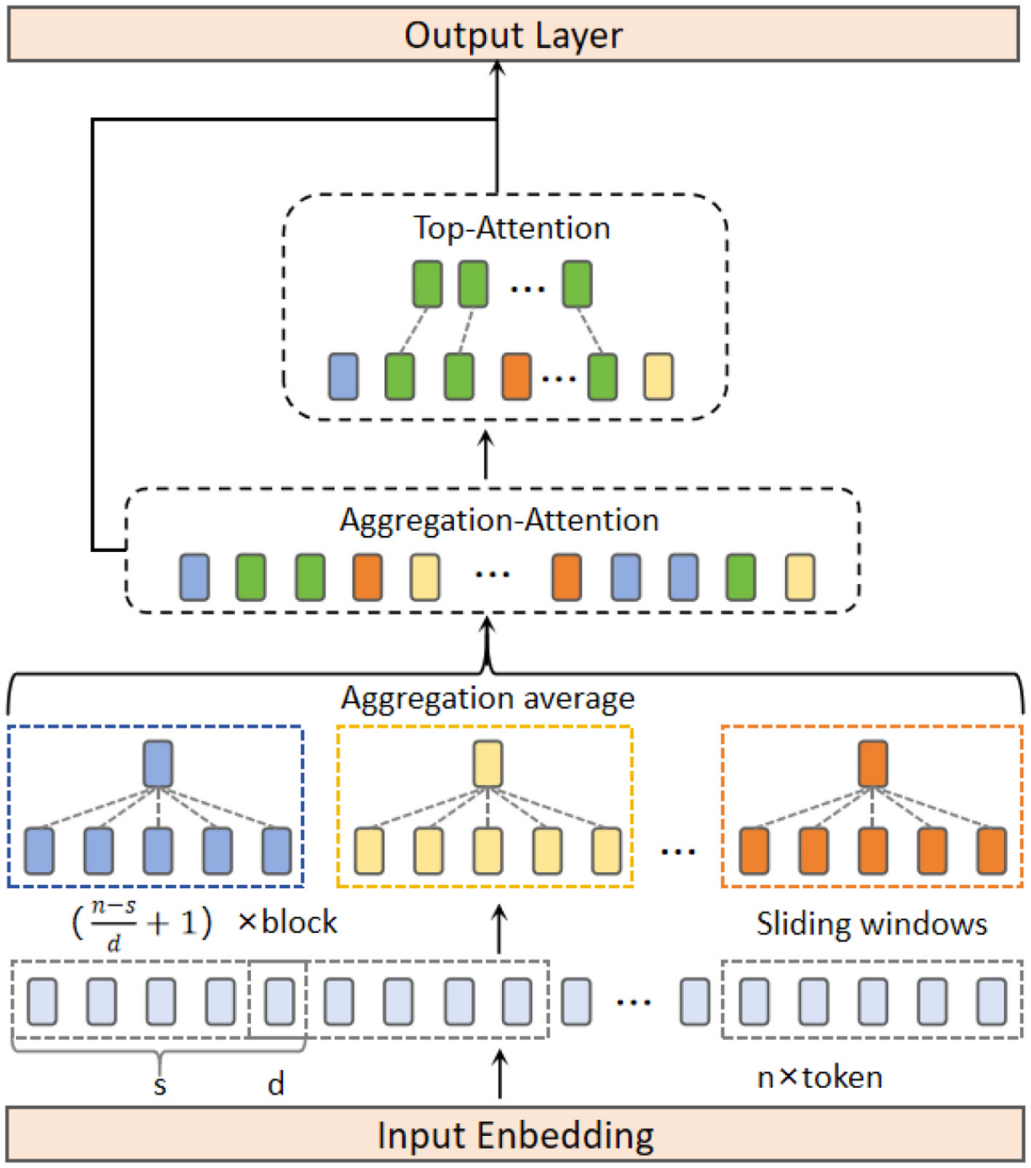
Structure of the SAT, which divides the sequence into multiple blocks using a sliding window, computes the mean attention of each block through Aggregation-Attention, and selects important blocks with the Top-Attention.

Global modeling approaches based on attention mechanisms face a trade-off between computational complexity and feature preservation (Vaswani et al., 2017): excessive focus on global dependencies can weaken local feature emphasis, while overemphasizing local features may result in loss of global context. The SAT overcomes the traditional challenge of balancing global and local features while improving EEG data processing efficiency. The SAT structure as shown in Figure 4.

#### 2.5.1 Sliding window

The sliding window partitions the input sequence into blocks, replacing the approach of feeding the entire sequence directly into subsequent layers. Using a sliding window of length *s*, the input sequence of length *n* is divided into *m* blocks with a stride of *d*, where $m=\frac{n-s}{d}+1$, *m*<*n*. When *d*≥*s*, the blocks do not overlap with each other. When *d*<*s*, there will be overlap between the blocks. Each block contains *s* consecutive tokens, producing a total of *n*_*s*_ = *m*×*s* tokens, *n*_*s*_>*n*. Compared to simply dividing the sequence into equal-sized, non-overlapping blocks, the sliding window approach increases the total number of tokens, which leads to higher computational cost. However, it partially compensates for the loss of inter-block connections. By adjusting the stride, the degree of overlap between blocks can be controlled, allowing the network to maintain block feature independence while enhancing global correlations. This design makes the overall network more flexible.

#### 2.5.2 Aggregation-attention

SAT introduces the concept of sparse attention (Beltagy et al., 2020; Huang et al., 2024), which eliminates the need for computing attention between every pair of positions. The *m* blocks divided by the sliding window are averaged respectively, and the average operation of *s* tokens within the blocks is aggregated into individual blocks to achieve the representation of global coarse-grained features. If all tokens within the blocks are expanded in sequence, the positional information of any token within a block can be represented as (*j*−1) × *d*+*i*. Let *K*_*j, i*_, *V*_*j, i*_ denote the key and value pairs of the *i*^*th*^ token in the *j*^*th*^ block, and the corresponding block-level key and value vectors be $K_{j}^{avg}$ and $V_{j}^{avg}$, respectively. These can be formalized as follows:


$$\begin{array}{l} K_{j}^{avg}=\frac{1}{s}\sum_{\left(j-1\right)\times d+1}^{\left(j-1\right)\times d+s}K_{j,i} \end{array}$$ (1)

$$\begin{array}{l} V_{j}^{avg}=\frac{1}{s}\sum_{\left(j-1\right)\times d+1}^{\left(j-1\right)\times d+s}V_{j,i} \end{array}$$ (2)

where 1 ≤ *j* ≤ *m* and 1 ≤ *i* ≤ *s*. Each query *Q*_*i*_ is dot-multiplied with all $K_{j}^{avg}$ to compute attention scores. Then, the weighted sum is obtained through $V_{j}^{avg}$, and the output of the aggregate attention path can be expressed as follows:


$$\begin{array}{l} Attn\left(Q_{i},K^{avg},V^{avg}\right)=Softmax\left(\frac{Q_{i}K^{avg^{T}}}{\sqrt{d_{k}}}\right)V^{avg} \end{array}$$ (3)

where *K*^*avg*^ and *V*^*avg*^ are the sets of $K_{j}^{avg}$ and $V_{j}^{avg}$, denoted as $K^{avg}=\left[K_{1}^{avg},\ldots ,K_{m}^{avg}\right]$ and $V^{avg}=\left[V_{1}^{avg},\ldots ,V_{m}^{avg}\right]$. By employing a block aggregation approach, the global features of the entire sequence can be obtained, and the computational complexity can be reduced from *O*(*n*^2^) to *O*(*n*·*m*), thereby enhancing the efficiency of sequence processing.

#### 2.5.3 Top-attention

Relying solely on aggregated blocks inevitably leads to feature information loss. Therefore, all blocks are selectively evaluated to retain the key feature blocks, minimizing accuracy loss during the modeling of long-range dependencies. Based on Aggregation-Attention, Top-k attention (Chen et al., 2023) is integrated. Specifically, the importance of each block is first evaluated using the attention scores obtained by the block, which avoids introducing new computational overhead. Then select the *k* highest blocks with the largest attention scores. In the EEG sequence scenario of this study, since the input sequence length to the Top-Attention module is relatively short (only *T*/75), the number of blocks *m* obtained after window partitioning is approximately 10, which is a small scale. At this point, the selection of *k* must balance sparsity and feature retention. Specifically, the introduction of sparse filtering in Top-Attention aims to focus on a small number of key blocks to reduce computational redundancy while preventing non-critical information from diluting the attention weights. When the input sequence itself is short and the total number of blocks is small, if *k* is set too large, the number of selected blocks will approach the total number of blocks, which is almost equivalent to no effective sparsity. In this case, Top-Attention degenerates into a near full-block computation mode, losing the advantage of reduced complexity through sparse selection. On the other hand, introducing too many non-key blocks causes the attention weights to become dispersed, weakening the model's ability to focus on core features. This contradicts the design logic of enhancing efficiency and precision through sparsity. Therefore, the value of *k* is generally chosen to be less than half of the total number of blocks *m*. Then generate a mask matrix *M* that depends on the attention score matrix *S*_*m*_. The selected k index positions are assigned a score of 1, while the remaining positions are assigned a score of −∞, forcing the weights of non-top blocks to approach zero. The attention score *S*_*k*_ can be expressed as:


$$S_{k}=\{\begin{array}{ll} M\frac{Q_{i}K^{avg^{T}}}{\sqrt{d_{k}}}, & \text{if }k\in \text{Top(row }j\text{)}\\ \;0\;, & \text{otherwise} \end{array}$$ (4)

where attention score matrix $S_{m}=\frac{Q_{i}K^{avg^{T}}}{\sqrt{d_{k}}}$. Subsequently, the s original tokens of the top blocks are recovered, and the position of the token corresponding to the *j*^*th*^ top block can be denoted as {(*j*−1) × *d*+1, …, (*j*−1) × *d*+*s*}. After the original tokens of the top blocks are unfolded in sequence, the total number of tokens *n*_*k*_ = *k*×*s*, *k*≪*m*. At this point, the original key-value pairs are restored to *K*^*top*^ and *V*^*top*^, and then the attention is calculated through *Q*_*i*_, which can be represented as:


$$\begin{array}{l} Attn\left(Q_{i},K^{top},V^{top}\right)=Softmax\left(\frac{Q_{i}K^{top^{T}}}{\sqrt{d_{k}}}\right)V^{top} \end{array}$$ (5)

where *K*^*top*^ and *V*^*top*^ are the sets of $K_{j,i}^{top}$ and $V_{j,i}^{top}$, denoted as $K^{top}=\left[K_{j,1}^{top},\ldots ,K_{j,k}^{top}\right]$ and $V^{top}=\left[V_{j,1}^{top},\ldots ,V_{j,k}^{top}\right]$. Top-Attention filters crucial blocks for computation, significantly reducing computational load compared to processing the original input sequence. By sparsely selecting k, complexity is further optimized to *O*(*n*·*k*), cutting computational redundancy while retaining key fine-grained features. Finally, SAT output combines results from Aggregation-Attention and Top-Attention, and is used in multi-head attention to achieve local and global perception of EEG information.

### 2.6 Classification

The classification module receives features processed by SAT. After passing through two fully connected layers with Dropout layers in between, the softmax function is used to compute the prediction probabilities for each class. The network is trained using a cross-entropy loss function L to optimize the difference between the model's predicted probability distribution and the true labels, and can be represented as follows:


$$\begin{array}{l} L=-\frac{1}{N_{b}}\overset{N_{b}}{\underset{i=1}{\sum }}\overset{M}{\underset{j=1}{\sum }}ylog\left(ŷ\right) \end{array}$$ (6)

where *N*_*b*_ denotes the batch quantity, *M* denotes the classification number, *y* represents the actual sample label, and ŷ represents the predicted sample label.

## 3 Experimental design

### 3.1 Datasets preparation

MI is the mainstream paradigm of EEG, allowing subjects to generate electrical signals in their brains merely by imagination. This paper takes the two datasets of BCI Competition IV 2a (Brunner et al., 2008) and BCI Competition IV 2b (Leeb et al., 2008) based on MI normal form as the experimental data support. Through EEG cross-session experiments, the effectiveness of the proposed APCformer decoding algorithm is verified. The specific details of the dataset are as follows.

#### 3.1.1 Dataset I

The BCI Competition IV 2a dataset was derived from the four types of motion imagination data of the left hand, right hand, feet and tongue of 9 subjects. Each subject performed two experiments, which were used respectively as the training set and the test set. Each Experiment consists of 6 groups of tests, and each group of tests contains 48 trials. At the beginning of the test (t = 0s), a fixed cross mark appears. When t = 2s, the subjects are guided up, down, left and right for 1.25s. The subjects need to complete the corresponding action imagination until the cross disappears at t = 6s, and then enter a 2s resting stage. The entire process lasts approximately 8s. In this paper, a 0.5–30Hz band-pass filter is applied to this dataset. For each trial, the 2–6s MI segment is retained. Based on the number of segments, the number of channels, and the number of samples, a single subject training dataset and test dataset are constructed with a size of [288, 22, 1,000].

#### 3.1.2 Dataset II

The BCI competition IV 2b dataset consists of two-class left-hand and right-hand MI-EEG data from 9 subjects. Each participant's data consists of 5 experiments, but the types of visual feedback are different. The first two experiments have no visual feedback and consist of 4 groups of trials, with 20 trials for each hand in each group. At the beginning of the experiment, a cross mark appears and then changes to a left or right arrow, which is displayed for about 1.25s. The participant needs to complete the imagination within 3 to 7s, and there will be a 1.5s rest period after that. The entire process lasts for 8.5 to 9.5s. The last three experiments have visual feedback and consist of 6 groups of trials, with 10 trials for each hand in each group. At the beginning of the experiment, a gray smiling face is displayed. At t = 3s, the participant is required to imagine moving the smiling face to the left or right. If correct, the smiling face turns green; if incorrect, it turns red and becomes a sad face. At t = 7.5s, the screen goes blank and there is a 1 to 2s rest period. In this paper, a band-pass filter retaining the 0.5–30Hz frequency band is applied. The 3–7s MI segment of each trial is extracted for subsequent analysis. Based on the number of segments, the number of channels, and the number of samples, a single-subject training dataset and test dataset are constructed with a size of [n, 3, 1,000], where n denotes the number of trials completed by each subject.

#### 3.1.3 Data preprocessing

EEG data can be represented as *X*∈ℝ^*C*×*T*^, where *C* denotes the number of EEG channels and *T* represents the number of sampling points along the time dimension. First, a bandpass filter is applied to extract the task-relevant frequency band of 0.5–30Hz from the EEG signals, eliminating noise and artifact components. Each MI-EEG fragment signal has independent temporal characteristics. If it is uniformly standardized across fragments or channels, it is easy to mask the baseline fluctuations of individual fragments and channel-specific differences. Therefore, Z-score standardization processing from fragment to channel is adopted one by one (Song et al., 2022; Zhao W. et al., 2024). The specific implementation is as follows: Let *x*_*n, c, t*_ denote the EEG signal at the *t*-th sampling point of channel *c* in segment *n*, and let *z*_*n, c, t*_ denote the standardized signal. For each segment *n* and each channel *c*, compute the mean μ_*n, c*_ over all sampling points, reflecting the local baseline level. It can be expressed as:


$$\begin{array}{l} \mu_{n,c}=\frac{1}{T}\overset{t_{n}}{\underset{t=1}{\sum }}x_{n,c,t} \end{array}$$ (7)

where *t*_*n*_ represents the total number of sampling points for a single fragment, ensuring that the mean calculation is limited to the current channel of the current fragment only. Then calculate the unbiased standard deviation σ_*n, c*_ of all sampling points and quantify the local amplitude dispersion, which can be expressed as:


$$\begin{array}{l} \sigma_{n,c}=\sqrt{\frac{1}{T-1}\overset{t_{n}}{\underset{t=1}{\sum }}{\left(x_{n,c,t}-\mu_{n,c}\right)}^{2}} \end{array}$$ (8)

where the denominator is taken as *T*−1 to reduce the statistical bias of the single-fragment sampling points. Then substitute the data *x*_*n, c, t*_ into the formula to obtain the standardized signal *z*_*n, c, t*_, which can be expressed as:


$$\begin{array}{l} z_{n,c,t}=\frac{x_{n,c,t}-\mu_{n,c}}{\sigma_{n,c}} \end{array}$$ (9)

Each channel of each segment transformed from the Z-score satisfies the distribution characteristics of a mean of 0 and a standard deviation of 1, thereby reducing the volatility and non-stationarity of the data.

#### 3.1.4 Data augmentation

EEG data training in small sample scenarios is highly prone to model overfitting, which seriously restricts the accuracy and generalization performance of the model. In this paper, the Segmentation and Reconstruction (S&R) technique is employed in the time domain to conduct data enhancement processing on EEG signals (Song et al., 2022; Zhao W. et al., 2024). The specific process is as follows: Before the model starts to learn the data features, the training data is segmented. The samples of [N, C, T] are divided by category, and the samples of each category are evenly divided into *Ns* sub-samples. First, determine the segmentation time window. Considering that the duration of the MI task process is 4 seconds and the sampling rate is 250Hz, it is necessary to ensure that the number of sampling points for each sub-sample in the segmentation is an integer. In this paper, *Ns* = 8 is set, that is, each sub-sample has 125 sampling points. Generally, there are not too many segmented samples, so that the reconstructed ones can contain complete EEG features related to MI. Then, the reconstruction operation is carried out. A sub-sample is randomly selected from each sample for recombination. A total of *Ns* of sub-samples are combined into new sample data. The sampling time points are restored to *T*. This process is repeated continuously until all the sub-samples that have been segmented in the samples have been recombined. However, during the reconstruction process, the principle of time sequence must be strictly followed. The arrangement order of sub-samples in the new sample should be consistent with the temporal sequence logic of the fragments in the original sample to avoid disrupting the inherent rhythm modulation rules of the EEG. The number of samples can be expanded without changing the physiological characteristics of the EEG signal. Subsequently, on the basis of achieving a multiple expansion of the sample size through segmentation and reconstruction, Gaussian noise *G*_*n*_ that follows a normal distribution is further injected into the new data. The fluctuation range of *G*_*n*_ is limited to the interval of [–1, 1], which neither masks the effective EEG features nor simulates the individual differences in EEG signals of different subjects, forcing the model to learn more robust features. Finally, the new samples were mixed with the original batch samples and randomly arranged in a shuffled order for joint training.

### 3.2 Experimental settings

The experiments were implemented using the PyTorch framework, and the hardware platform used was an NVIDIA GTX 1660 SUPER GPU. The training process utilized five-fold cross-validation, with a total of five training rounds. Each round had a maximum of 100 training epochs. We adopted an within-session experimental evaluation protocol, which can improve data utilization and result stability, and focus on verifying the effectiveness of all model structures. For each session of the dataset, all samples are randomly shuffled and divided into a training set and a test set with a ratio of 80% and 20%. Meanwhile, 20% of the training set is further randomly divided as the validation set during the training process. The model that performs best on the validation set is chosen for the evaluation of the test set. The final evaluation metric is the average result obtained from multiple tests on the test set. The batch size for the training data was set to 32, and the Adam optimizer with a cross-entropy loss function was employed to guide the model in minimizing prediction errors. The learning rate was set to 0.001, and a cosine annealing scheduler was used to gradually reduce the learning rate and optimize training performance.

### 3.3 Performance metrics

We adopts two key indicators, accuracy rate (Herman et al., 2008) and Kappa coefficient (Chen et al., 2020), to comprehensively evaluate the performance of the model. Accuracy is the most direct standard for measuring the model's performance, reflecting the proportion of correct judgments among all predicted samples. Its calculation method can be expressed as:


$$\begin{array}{l} Accuracy=\frac{TP+TN}{TP+TN+FP+FN} \end{array}$$ (10)

where *TP* represents the true example, *TN* represents the true negative example, *FP* represents the false positive example, and *FN* represents the false negative example. The accuracy value ranges from 0 to 1. In the MI-EEG decoding task, its numerical value directly corresponds to the success rate of intention recognition. The Kappa coefficient is an indicator for measuring classification accuracy. Its core value in EEG decoding lies in evaluating the robustness of the model. Its calculation method can be expressed as:


$$\begin{array}{l} Kappa=\frac{P_{o}-P_{e}}{1-P_{e}} \end{array}$$ (11)

where *P*_*o*_ is the observed accuracy, *P*_*e*_ represents the consistency probability due to randomness. In addition, the *P*-value was evaluated using the Wilcoxon signed rank test (Wilcoxon, 1992) to assess the significance of the statistical differences between APCformer and other baseline models. Among them, *P*-value < 0.01 indicates that the difference is highly statistically significant, *P*-value < 0.05 indicates that the difference is statistically significant, and *P*-value > 0.05 indicates that there is no statistically significant difference.

## 4 Experimental result

### 4.1 Training process

**Figure 5:**
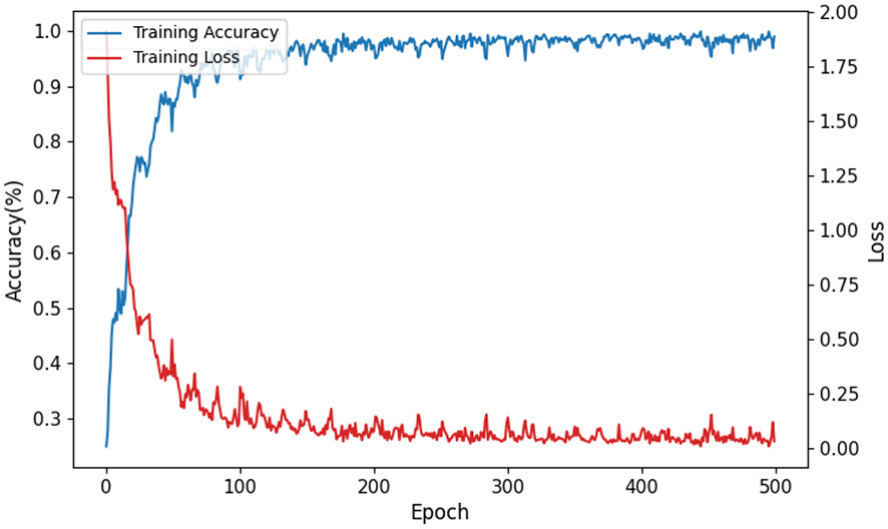
The dynamic variation curve of accuracy and loss values of APCformer on the training set.

Figure 5 shows the dynamic variation curve of APCformer after continuous learning for 500 epochs on the A03 subjects of dataset I. During the first 100 epochs, the accuracy increases rapidly as the training progresses. After 150 epochs, the accuracy curve quickly converges and stabilizes. Corresponding to the accuracy trend, the loss curve also shows an efficient decrease in prediction error, with a sharp reduction in the early training stages. As the training continues, the loss value ultimately approaches 0. Overall, no signs of overfitting are observed, providing strong evidence that APCformer's network optimization is effective.

### 4.2 Baseline comparison

We selected nine commonly used MI-EEG decoding methods and compared them with APCformer. To ensure the fairness of the comparison, all models were re-evaluated. These models had significant differences in experimental conditions from those in the original literature. For instance, the filtering range of EEG signals in this study was different from that in the original literature, as detailed in Section 3.1.3. Compared with the average accuracy rates reported in the original literature, most of the models evaluated in this study achieved similar results. In the experiments of this article, all models were evaluated for performance under the same experimental conditions, using the same dataset, preprocessing procedure, dataset division ratio, data augmentation strategy, and training strategy. The selected benchmark models directly called their publicly available official codes, and all hyperparameters followed the default optimal settings stated in the original literature.

1) DeepConvNet (Schirrmeister et al., 2017): A network based purely on CNN, which captures relevant features through convolution and compresses redundant information along the time dimension, though its long-range dependency modeling is relatively weak.

2) EEGNet (Lawhern et al., 2018): A lightweight network designed for small EEG datasets, which decouples spatial and temporal features using depthwise separable convolutions, effectively handling high-dimensional EEG data and low signal-to-noise ratio (SNR) issues.

3) ITNet (Salami et al., 2022): A network that extracts characteristics through the parallel use of Inception modules and dilated causal convolutions. It perceives both spatial and temporal information of EEG signals at multiple scales, achieving higher performance with lower complexity.

4) Conformer (Song et al., 2022): A compact CNN-Transformer network that uses 1D convolutions to extract local features and self-attention to reveal global correlations, balancing performance and efficiency.

5) CTNet (Zhao W. et al., 2024): A CNN-Transformer network that uses a convolutional structure similar to EEGNet to extract local spatiotemporal features, combined with multi-head attention to capture global dependencies in the features.

6) ADFCNN (Tao et al., 2023): A dual-scale parallel network that uses two-scale convolutions to jointly identify EEG features, applying self-attention to enhance the flexibility of feature fusion, with good application performance.

7) MSFCNNet (Yang and Liu, 2024): A multi-scale hierarchical network that captures local details through multi-scale cascading and enhances long-range dependencies through multi-head attention, achieving better accuracy compared to single-scale networks.

8) MSCFormer (Zhao et al., 2025b): A multi-scale convolutional Transformer hybrid architecture, which employs multi-branch multi-scale CNN to extract local spatiotemporal features and combines the Transformer encoder to model global dependencies, effectively improves the classification performance of MI-EEG.

9) TCANet (Zhao et al., 2025a): A progressive feature learning network, which extracts and compresses features through multi-scale convolution and temporal convolution, stacks multi-head attention to enhance global dependencies, and achieves more accurate MI-EEG classification on public datasets.

Table 2 presents a comparison of the accuracy of various models on Dataset I. While CNN-based methods such as DeepConvNet, EEGNet, and ITNet perform well in local feature extraction, they fail to fully consider the global correlations of EEG signals, limiting decoding accuracy. Their accuracies are 74.22% (*p* < 0.01), 76.39% (*p* < 0.01), and 77.66% (*p* < 0.05), respectively. Among these, ITNet achieves the highest accuracy due to its multi-scale structure, which provides a broader perceptive range, demonstrating that incorporating multi-scale features can improve network performance. In contrast, Transformer-enhanced models such as Conformer and CTNet have advantages in temporal processing, with accuracies increasing to 78.57% (*p* < 0.01) and 81.92% (*p* < 0.05), respectively. However, they neglect the importance of different feature levels and fail to focus on fine-grained features. The hybrid models that combine multi-scale CNN and Transformer architectures, such as ADFCNN, MSFCNNet, MSCFormer and TCANet, they all further enhance the MI-EEG decoding accuracy, reaching 81.42% (*p* < 0.05), 83.32% (*p* < 0.05), 84.26% (*p* < 0.05), and 84.78% (*p* < 0.05) respectively. However, they lack effective cross-scale correlations, and thus still struggle to strike a balance between global and local relationships. APCformer addresses these shortcomings by effectively combining local and global features, achieving the best performance with an average accuracy of 85.53%, outperforming all other models.

**Table 2:** Comparison of classification accuracy (%) on Dataset I.

**Methods**	**A01**	**A02**	**A03**	**A04**	**A05**	**A06**	**A07**	**A08**	**A09**	**Avg**	**Std**	**Kappa**	***P*-value**
DeepConvNet	86.66	56.81	88.72	67.44	56.36	59.87	83.20	81.55	87.34	74.22	13.90	0.66	0.0039
EEGNet	86.13	62.27	91.58	69.44	61.31	55.77	89.42	82.60	89.03	76.39	14.12	0.69	0.0039
ITNet	91.66	61.39	88.78	71.32	73.66	61.54	78.16	79.85	92.62	77.66	11.88	0.70	0.0173
Conformer	**92.63**	65.21	93.17	69.35	60.13	63.42	91.24	83.79	88.22	78.57	13.81	0.71	0.0078
CTNet	88.41	76.08	94.56	76.60	67.33	75.67	86.62	80.95	91.09	81.92	8.83	0.76	0.0195
ADFCNN	86.52	77.28	91.76	75.31	73.26	61.70	91.52	83.34	92.07	81.42	10.38	0.75	0.0117
MSFCNNet	88.76	78.11	93.37	71.44	74.03	72.62	90.45	87.92	**93.18**	83.32	9.14	0.78	0.0195
MSCFormer	91.21	**78.49**	94.62	76.39	75.45	70.73	92.61	87.43	91.38	84.26	8.96	0.79	0.0195
TCANet	91.83	72.62	94.75	76.06	**77.23**	**76.18**	93.57	89.74	91.03	84.78	8.98	0.80	0.0195
**APCformer**	91.66	76.85	**95.11**	**78.27**	75.98	74.50	**94.13**	**90.88**	92.41	**85.53**	**8.81**	**0.81**	-

In the table, the best data of models are indicated in bold. Avg represents the average accuracy rate of the 9 subjects. Std is the standard deviation. The *P*-value was calculated using the Wilcoxon signed rank test.

**Figure 6:**
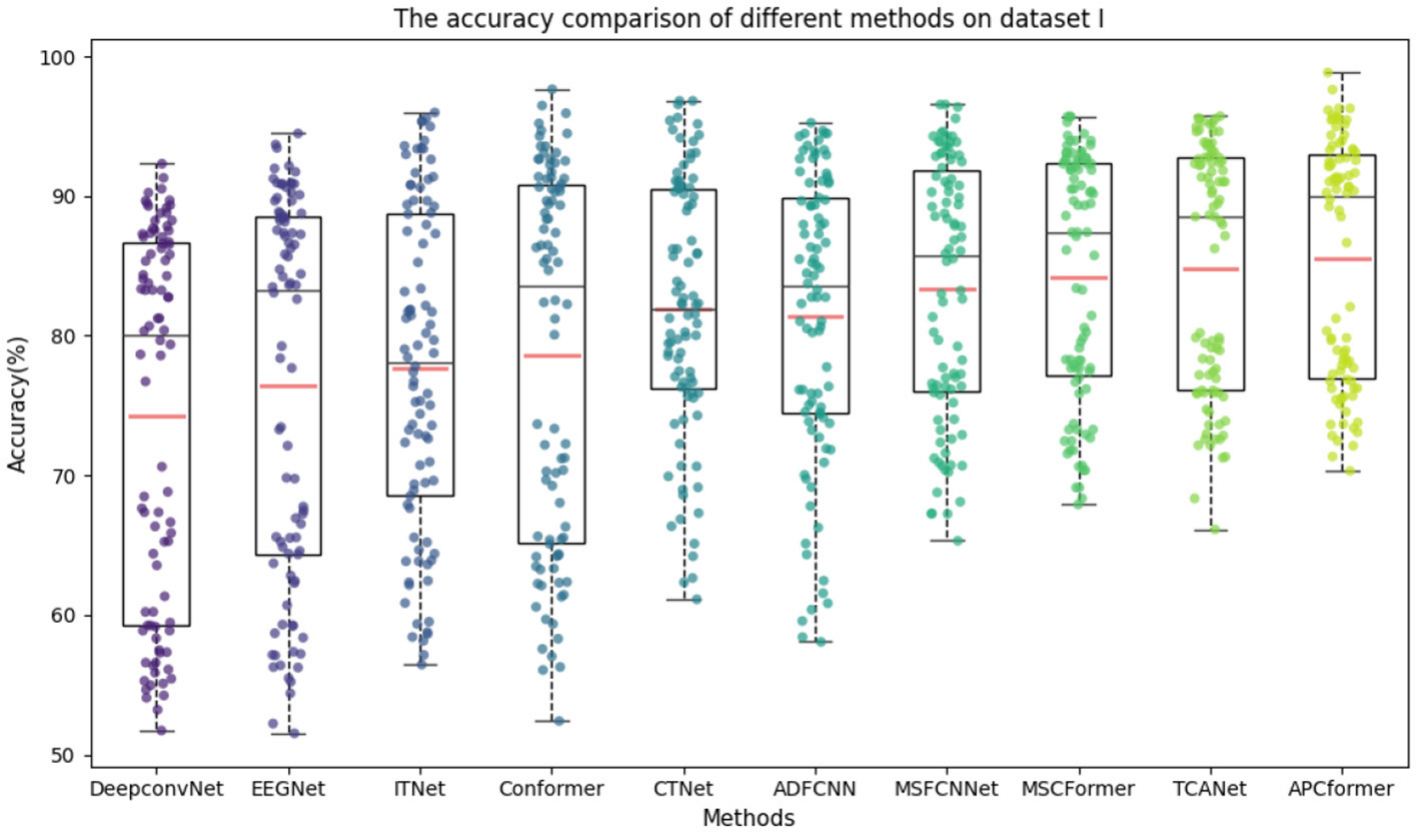
A box-shaped scatter plot comparing the accuracy of the same model on all subjects in dataset I. Among them, the box range represents the standard deviation of all participants under different models, and the red line in the box represents the average value obtained by the corresponding model.

To further evaluate the performance and robustness of the APCformer against baseline models, box plots of classification accuracies for different models were created. Box plots help analyze horizontal differences across data categories and reveal data dispersion, outliers, and distribution variations. As shown in Figure 6, in the box plot, the box range, red line, and median line represent the standard deviation, mean, and median of classification accuracies across all participants for each model. Other baseline models show lower box positions and median lines, with dispersed data points and large accuracy fluctuations. In contrast, the median line of APCformer is higher and the data points are more densely distributed, indicating that it has greater stability.

Table 3 presents the comparison of APCformer on Dataset II. Compared to other methods, APCformer demonstrates a significant advantage, with average accuracy improvements of 8.05% (*p* < 0.05), 7.52% (*p* < 0.01), 5.7% (*p* < 0.05), 3.68% (*p* < 0.05), 4.63% (*p* < 0.01), 2.6% (*p* < 0.05), 0.83% (*p* < 0.05), 0.88% (*p* < 0.05), and 0.34% (*p* < 0.05), achieving the highest accuracy of 89.15% among all methods. Additionally, APCformer outperforms other comparison methods in terms of accuracy for most subjects across both datasets. These comparative experimental results indicate that APCformer surpasses other methods in key evaluation metrics, confirming its effectiveness and reliability in EEG decoding tasks.

**Table 3:** Comparison of classification accuracy (%) on Dataset II.

**Methods**	**B01**	**B02**	**B03**	**B04**	**B05**	**B06**	**B07**	**B08**	**B09**	**Avg**	**Std**	**Kappa**	***P*-value**
DeepConvNet	76.33	60.44	70.54	93.81	93.63	75.53	82.86	93.04	83.65	81.09	11.53	0.75	0.0117
EEGNet	79.22	57.59	68.20	95.54	92.48	75.06	85.11	92.34	89.13	81.63	12.76	0.76	0.0078
ITNet	84.62	65.10	66.39	91.94	95.55	82.10	81.26	92.14	91.88	83.44	11.18	0.78	0.0195
Conformer	81.31	67.15	69.84	97.06	90.65	88.48	90.92	94.24	89.59	85.47	10.55	0.81	0.0391
CTNet	78.64	66.62	72.52	95.18	95.34	83.87	90.84	86.46	91.37	84.52	10.14	0.79	0.0078
ADFCNN	81.07	69.88	70.20	97.32	96.41	87.85	90.97	94.02	91.12	86.55	10.51	0.82	0.1289
MSFCNNet	84.12	71.73	76.61	98.74	94.94	**92.93**	87.19	93.73	**94.86**	88.32	9.2	0.84	0.4961
MSCFormer	84.27	73.82	75.64	97.08	**96.67**	89.25	91.61	94.81	91.23	88.26	8.63	0.84	0.0391
TCANet	86.54	72.35	78.19	98.23	93.69	89.94	92.28	**95.75**	92.34	88.81	8.48	0.85	0.0391
**APCformer**	**88.95**	**76.71**	**78.22**	**98.86**	94.96	87.80	**92.75**	90.32	93.75	**89.15**	**7.41**	**0.86**	-

In the table, the best data of models are indicated in bold. Avg represents the average accuracy rate of the 9 subjects. Std is the standard deviation. The *P*-value was calculated using the Wilcoxon signed rank test.

### 4.3 Ablation study

#### 4.3.1 Compare the influence of each component on the model

To verify the functions of each core module of APCformer, taking the complete APCformer model as the benchmark, the MS, AFR, PAE, and SAT modules were removed successively on dataset I. The comparison results of each variant are shown in Table 4. The average accuracy rate and Kappa coefficient of the complete model are the best, and each module or function plays an important role. Among them, after removing the SAT module, the average accuracy rate dropped to 80.86%, with the largest decline being 4.67%, highlighting its superior performance in global and local feature processing. After removing the SAT and then the PAE module, the average accuracy rate dropped to 79.18%, a decrease of 1.68%. This is because the PAE module enhances the temporal correlation of deep features through learnable position encoding. After removal, the model has difficulty capturing the dynamic evolution of EEG, which affects the classification performance. The interactive structure of the AFR module can screen out key features and suppress redundancy. After removal, the multi-scale shallow features lack optimization, and the effective information is masked, further reducing the average accuracy rate to 77.66%, a decrease of 1.52%. The effect is not good when only multi-scale convolutional modules are used because they only stop at extracting shallow local features. However, it still outperforms the single-scale structure of EEGNet (76.39%), indicating that the multi-scale structure can effectively improve the prediction accuracy. In addition, the data augmentation strategy reduces the model's sensitivity to interference by expanding sample diversity, enhances the model's generalization ability, and increases the average accuracy of the model by 2.76%. In the AFR module, by comparing the variant models without interaction functions, the contribution of the interaction mechanism to the model performance was verified. With the support of the non-interaction function, the model accuracy decreased by 1.32% (84.21%), indicating that the interaction function can effectively promote the fusion of multi-scale features and enhance the cross-scale correlation between branches. While maintaining the depth and quantity of attention, by comparing the performance differences between using the standard Transformer encoder model and using the SAT model, the experimental results show that the SAT performs better in capturing the temporal dependencies of EEG signals and integrating global and local features. The classification accuracy is 1.74% higher than that using the standard Transformer encoder. Based on the comprehensive experimental results, it is proved that APCformer can enhance the feature extraction ability and prediction accuracy through module collaboration.

**Table 4:** Comparison of classification accuracy (%) of ablation study.

**STConv**	**AFR**	**PAE**	**SAT**	**DA**	**w/o-Int**	**Trans**	**Avg**	**Std**	**Kappa**
✓	✓	✓	✓	✓	−	−	**85.53**	**8.81**	**0.81**
✓	✓	✓	−	✓	−	−	80.86	9.08	0.74
✓	✓	−	−	✓	−	−	79.18	10.88	0.72
✓	−	−	−	✓	−	−	77.66	9.97	0.70
✓	✓	✓	✓	−	−	−	83.17	9.23	0.78
✓	−	✓	✓	✓	✓	−	84.21	9.03	0.79
✓	✓	✓	−	✓	−	✓	83.79	9.18	0.78

Among them, DA is for adding data enhancement processing, w/o-Int indicates the AFR module that does not use the interaction mechanism, and Trans represents the standard transformer encoder. Avg represents the average accuracy rate of the 9 subjects, √ indicates the use of this module component, and − indicates not using it. The best data of models are indicated in bold.

#### 4.3.2 Comparison of confusion matrices

**Figure 7:**
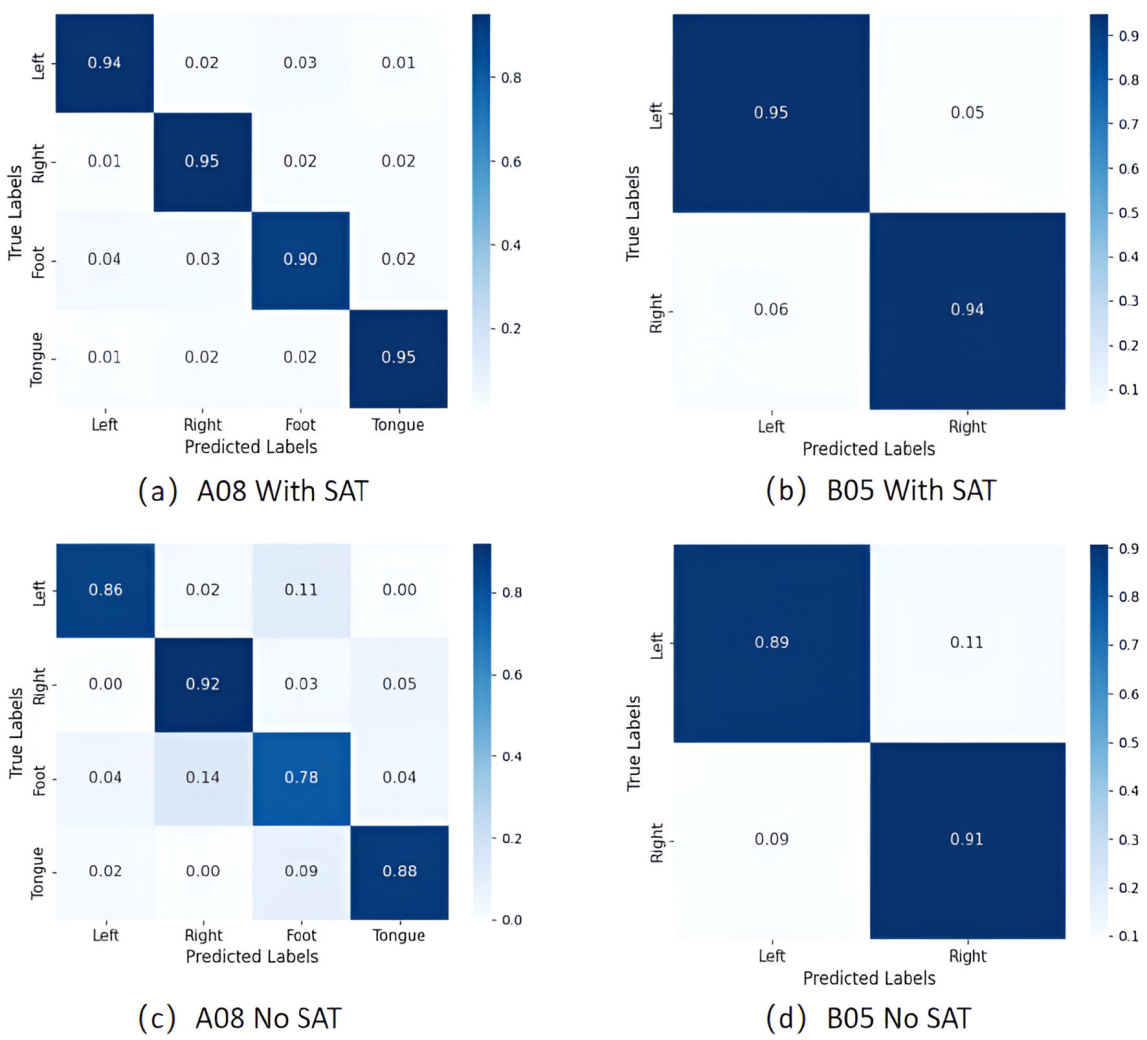
The confusion matrix of the classification results of APCformer on the two datasets: **(a)** Confusion matrix of APCformer with the SAT module for subject A08 in Dataset I; **(b)** Confusion matrix of APCformer with the SAT module for subject B05 in Dataset II; **(c)** Confusion matrix of APCformer without the SAT module for subject A08 in Dataset I; **(d)** Confusion matrix of APCformer without the SAT module for subject B05 in Dataset II. Among them, the horizontal axis represents the predicted labels, while the vertical axis represents the true labels. The classification accuracy can be intuitively observed from the color depth.

The confusion matrix can visually display the comparison results between the actual labels and the predicted labels, covering the proportion of correct and incorrect classifications. Figure 7 exhibits the confusion matrices of classification results for two datasets. It is evident that the diagonal elements are significantly higher than the others, indicating excellent predictive performance of the APCformer. From the dataset perspective, subject A08 in Dataset I includes four types of MI tasks: left hand, right hand, leg, and tongue. In Figure 7a, when the SAT module is included, the recognition accuracies for the right hand and tongue are the highest, both reaching 95%. The accuracy for the left hand task is slightly lower but still reaches 94%. The prediction accuracy for the leg task is the lowest among the four classes, though it still achieves 90%. In contrast, in Figure 7c, when APCformer operates without the SAT module, the prediction accuracy for the imagined leg movement drops to only 78%, with an error rate as high as 22%, making it the weakest performance among the four classes. Subject B05 in Dataset II involves two classes of tasks: left hand and right hand. In Figure 7b, with the SAT module, the recognition accuracies for the left and right hands reach 95% and 94%, respectively, whereas in Figure 7d, without the SAT module, the accuracies decrease to 89% and 91%. Notably, the left hand accuracy improves by 6% when the SAT module is included. These results indicate that even in tasks with different numbers of classes, the SAT module can still enhance classification stability by optimizing spatial feature weighting and reducing inter-individual variability within the same MI class.

In terms of overall performance, when the SAT module is included, as shown in Figures 7a, b, the average classification accuracy of the model on dataset I A08 and on dataset II B05 reaches 93.5% and 94.5%, respectively. Compared with the time without the SAT module, 86% in Figure 7c and 90% in Figure 7d have increased by 7.5% and 4.5% respectively. Especially in comparing the prediction accuracy of leg movements on A08 in dataset I and that of left hand movements on B05 in dataset II, it further confirms the significant improvement of overall performance brought by the SAT module. Among them, the prediction accuracy of leg movements has improved most significantly after the SAT module was added, with an increase of 12%. This difference is directly related to the physiological structural characteristics of the leg MI-EEG signal and the design advantages of the SAT module. On the one hand, the MI-EEG signals in the human body mainly originate from the motor cortex of the brain. The nerve conduction distance of leg movements is farther than that of other movements, the signal is weaker, and the proportion of noise is higher. On the other hand, the SAT module enhances the spatial feature discrimination of EEG signals, and its sequence modeling capability improves the capture of the spatiotemporal dynamics of EEG signals. It can better identify the more subtle temporal features in the movements of different parts of the brain, enabling the originally weaker and more complex leg EEG patterns to be decoded more clearly It demonstrated excellent classification accuracy and robustness in the EEG decoding task.

### 4.4 Model parameter sensitivity analysis

#### 4.4.1 Comparison of frequency bands and channel numbers

To investigate the impact of EEG frequency bands and the number of channels on the performance of APCformer, five frequency bands were configured: 0.5–8Hz, 8–12Hz (μ rhythm), 13–30Hz (β rhythm), 8–30Hz, and 0.5–30Hz. The electrode placement scheme is based on the central core electrodes C3, Cz, and C4 (Ke et al., 2024). A hierarchical channel expansion strategy is employed, gradually extending the coverage outward from the central region. Based on this approach, five electrode channel combinations are constructed for evaluation. The specific configurations are as follows:

- 3 channels: C3, Cz, C4
- 9 channels: Adds Fz, Pz, C1, C2, FCz, CPz to the 3-channel set
- 13 channels: Adds FC1, FC2, CP1, CP2 to the 9-channel set
- 17 channels: Adds FC3, FC4, CP3, CP4 to the 13-channel set
- 22 channels: All available EEG channels

**Figure 8:**
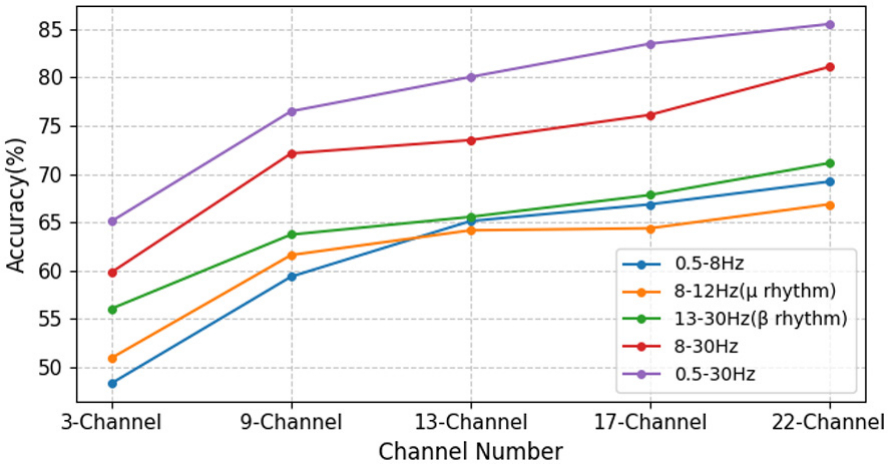
The influence of different EEG frequency bands and channel numbers on the model for all subjects in dataset I. Among them, the horizontal axis represents the number of EEG channels, and the legend indicates the selected EEG frequency band range.

As shown in Figure 8, across all frequency bands, classification accuracy consistently improves with an increasing number of EEG channels. Notably, the accuracy improvements are most pronounced for the 0.5–8Hz, 8–30Hz, and 0.5–30Hz bands, indicating that incorporating more channels provides richer EEG information, which positively contributes to model performance. When using fewer channels (3 or 9 channels), the gains from broader frequency bands are particularly noticeable. This may be attributed to the fact that critical EEG activity is mainly concentrated in the central motor areas and a few surrounding regions. Once the channel count reaches 13, the rate of accuracy improvement slows, suggesting that most of the relevant information has already been captured. Although additional peripheral channels continue to contribute, their impact diminishes, reflecting a saturation effect. Nonetheless, the overall trend remains upward. Comparing across all frequency bands, the 0.5–30Hz and 8–30Hz bands significantly outperform narrower bands. For instance, with 22 channels, the 0.5–30Hz band achieves an accuracy of 85%, which is markedly higher than the 65% accuracy observed for the 8–12Hz band. This suggests that wider frequency bands may contain more comprehensive and informative neural signals, while narrower bands are comparatively limited in their representational capacity. In summary, the experimental results demonstrate that the performance of APCformer improves significantly with an increased number of channels and broader frequency bands. This indicates that appropriately integrating more spatial channels and wider spectral EEG information can effectively enhance the model's decoding accuracy.

#### 4.4.2 Comparison of convolution kernel sizes

**Figure 9:**
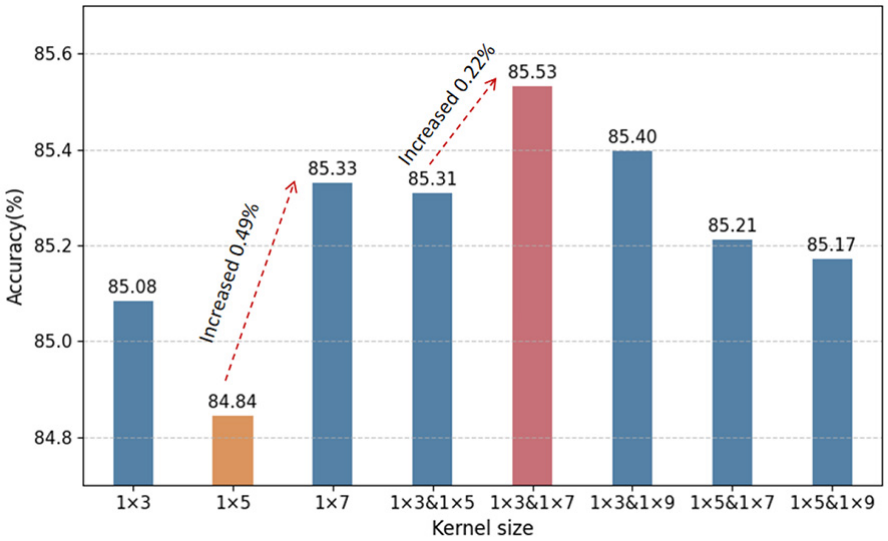
Compare the influence of PAE with convolution kernels of different scales on the model accuracy on all subjects in dataset I. Among them, the vertical axis represents the model accuracy, while the horizontal axis indicates the convolution kernel scale combinations used in the PAE module. For example, 1 × 3 refers to the single-scale 1 × 3 convolution kernel, and 1 × 3&1 × 5 represents a multi-scale combination of 1 × 3 and 1 × 5 convolution kernels. The red dashed line and the labeled “Increased × × %” quantify the accuracy improvement brought by the PAE module. The numbers above each bar correspond to the specific accuracy for each convolution kernel combination.

An appropriate kernel size can significantly improve model performance. To evaluate the impact of different convolution scales within the PAE module, eight configurations were compared, including single-scale kernels (1 × 3, 1 × 5, 1 × 7) and parallel multi-scale combinations (1 × 3 & 1 × 5, 1 × 3 & 1 × 7, 1 × 3 & 1 × 9, 1 × 5 & 1 × 7, 1 × 5 & 1 × 9). As shown in Figure 9, among the single-scale structures, the 1 × 5 kernel achieved an accuracy of only 84.84%, while the 1 × 7 kernel achieved 85.33%, indicating relatively better performance for larger receptive fields. Overall, single-scale configurations did not demonstrate a clear advantage. The 1 × 3 & 1 × 7 combination achieved the highest accuracy at 85.53%, outperforming the 1 × 3 and 1 × 7 kernels by 0.45% and 0.2%, respectively, and exceeding the lowest-performing 1 × 5 kernel by 0.69%. This highlights that parallel structures with complementary receptive fields can enhance feature extraction. However, not all kernel combinations yielded performance gains. For example, the combinations of 1 × 3 & 1 × 5, 1 × 5 & 1 × 7, and 1 × 5 & 1 × 9 all resulted in lower accuracy than the single 1 × 7 configuration, indicating that arbitrary kernel pairings may not be beneficial. The choice of kernel sizes must be deliberate, as effective scale configurations can improve model performance. In summary, the results suggest that PAE modules using multi-scale parallel convolutional structures can better coordinate feature extraction, enhancing the diversity and richness of learned representations.

#### 4.4.3 Attention parameters analysis

**Figure 10:**
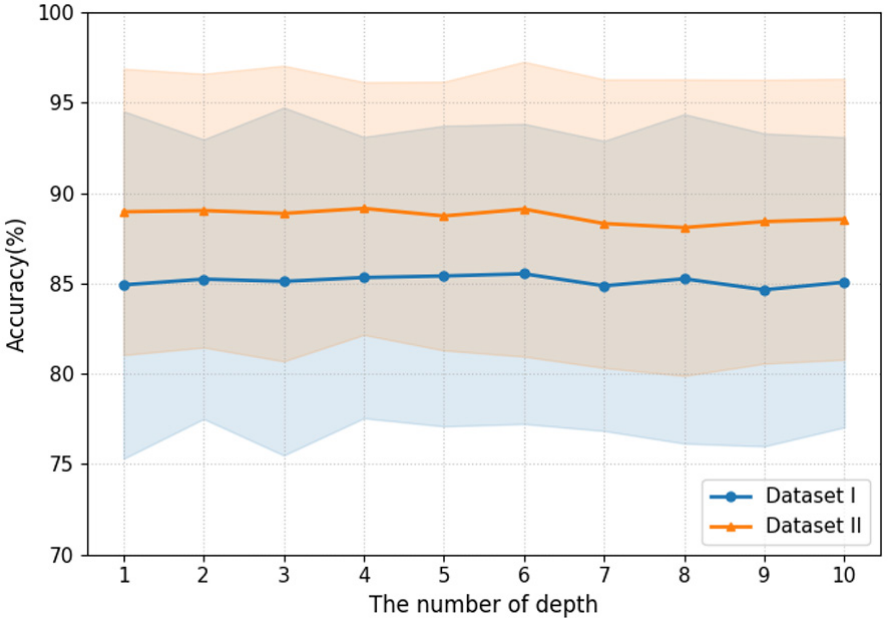
The influence of different attention layer depths on the accuracy of APCformer on datasets I and II. Among them, the horizontal axis represents the number of levels of attention, ranging from 1 to 10 levels.

The attention layer depth (*N*) and the number of attention heads (*h*) are important parameters in the multi-head attention mechanism (MHA), and their selection can influence the model's fitting capability to a certain extent. Increasing the attention layer depth provides the model with stronger feature extraction ability. As the number of layers increases, the model gradually transforms simple local correlations in the input sequence into higher-level global contextual representations, thereby capturing more complex sequential patterns and long-range dependencies. As shown in Figure 10, we investigate the effect of depth on the APCformer by gradually increasing the number of attention layers. The experiments are conducted on all subjects from Dataset I and Dataset II, and the overall average accuracy is reported. A total of ten configurations of attention layer depth are tested. From the figure, it can be observed that increasing the number of attention layers from 1 to 10 does not cause significant changes in the average model accuracy on either Dataset I or Dataset II, the overall accuracy remains relatively stable. Specifically, on Dataset I, the highest average accuracy is achieved when *N* = 6, while the lowest average accuracy occurs when *N* = 9, with the difference between the two being less than 1%. Similarly, on Dataset II, the highest average accuracy is obtained when *N* = 4, which is only 1.06% higher than the lowest accuracy achieved when *N* = 8. These results indicate that the number of attention layers has a relatively small effect on model performance in both Dataset I and Dataset II.

**Figure 11:**
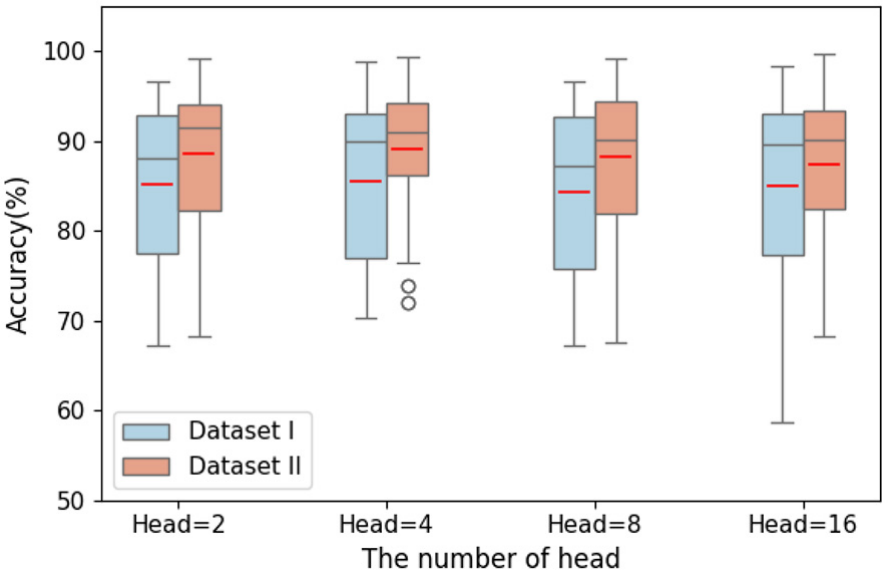
The influence of different numbers of attention heads on the accuracy of APCformer was adopted on datasets I and II. Among them, the horizontal axis represents the number of heads of attention, and four groups of heads with Head = 2, 4, 8, and 16 are selected.

The number of attention heads enables the model to capture correlations among different subspaces of the sequence in parallel. Different attention heads spontaneously focus on distinct dimensions of the input sequence, allowing the model to interpret sequence information from multiple perspectives and thereby enhancing its fine-grained understanding of complex patterns. As shown in Figure 11, the effect of varying the number of attention heads on APCformer's performance is further explored. The experiments are conducted on all subjects from both Dataset I and Dataset II, and the overall average accuracy is reported. Four configurations of attention heads are tested, with *h* = 2, 4, 8, and 16. From the figure, it can be observed that the influence of the number of attention heads on APCformer is generally consistent with that of layer depth. Although there are slight fluctuations in overall average accuracy, the differences between configurations are not significant. For instance, on Dataset I, the difference in average accuracy is 1.26%, while on Dataset II, it is 1.72%. It is also evident that the APCformer model achieves the best performance when equipped with *h* = 4 attention heads. Similarly, this indicates that the number of attention heads has a relatively minor impact on model performance in both Dataset I and Dataset II. Combining the experimental results regarding the effects of attention layer depth and attention head number on APCformer's performance, it can be concluded that these two parameters are not the key factors determining APCformer's performance. In contrast, the computational cost of the attention mechanism increases linearly with both the number of layers and the number of heads. Thus, excessively large values for these parameters would increase computational complexity and consume additional computing resources.

#### 4.4.4 Sliding window parameter analysis

**Figure 12:**
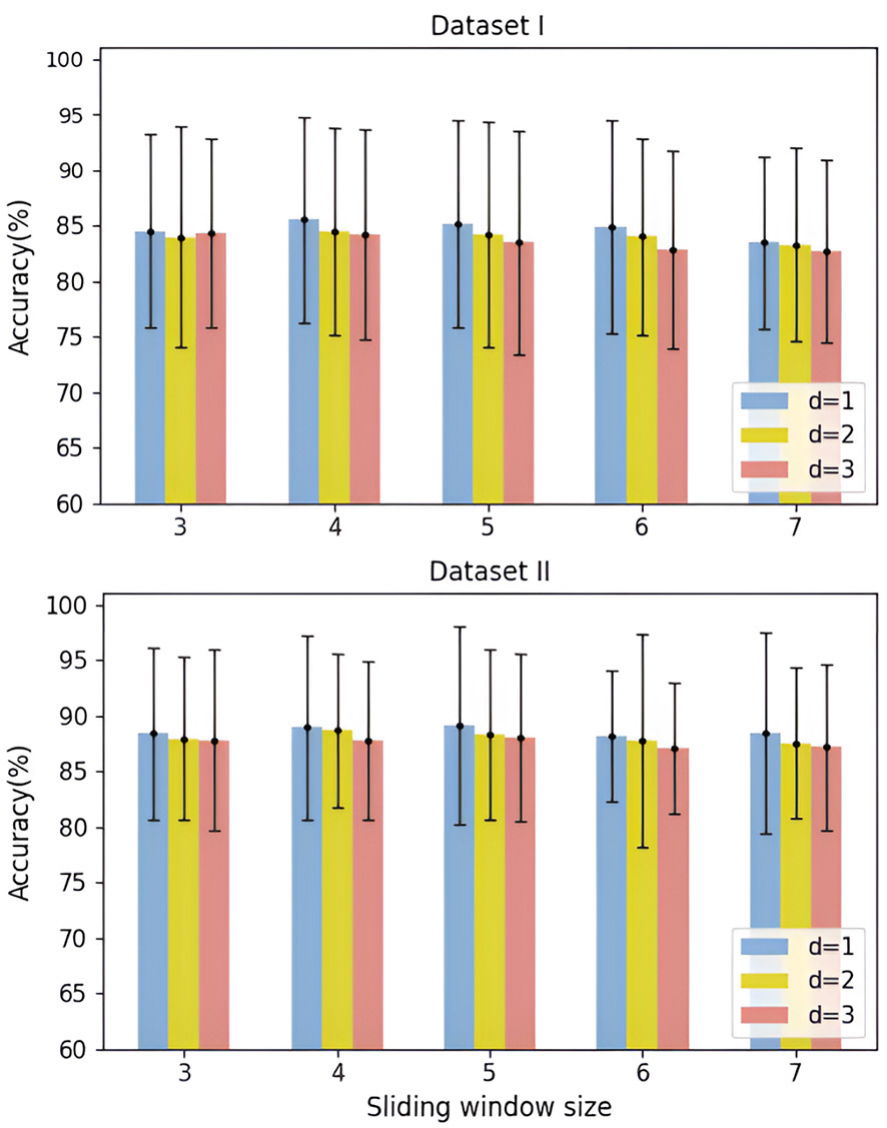
Compare the effects of the sliding window and sliding distance in the SAT module on the accuracy of APCformer on all subjects in dataset I. The upper and lower graphs show the results obtained by APCformer on Dataset I and Dataset II, respectively. The horizontal axis represents the sliding window size used in the SAT module, and the legend indicates the sliding stride of the window.

The sliding window size *s* and stride *d* used in the SAT module directly affect the number of blocks extracted from the input sequence, thereby influencing the model's ability to capture temporal patterns. The input sequence length to SAT is fixed, as determined by the preceding modules. In this experiment, five window sizes (ranging from 3 to 7) and three stride values (1, 2, 3) were tested. As shown in Figure 12, on Dataset I, the highest accuracy was achieved when the window size was *s* = 4 and stride *d* = 1; on Dataset II, the optimal configuration was *s* = 5, *d* = 1. Further analysis reveals that configurations with *s* = 4 and *s* = 5 consistently outperformed other settings, and all optimal results were observed when *d* = 1. This suggests that a smaller stride is more conducive to capturing the temporal variations in EEG sequences. As both window size and stride increase, the number of blocks decreases. Consequently, the model's accuracy tends to decline, especially in extreme configurations such as *s* = 7, *d* = 3, where fewer blocks are generated. In this case, large window and stride settings lead to excessive aggregation of temporal features, causing loss of critical information and reduced decoding precision. Conversely, although a window size of *s* = 3 yields the highest number of blocks, its limited temporal coverage may result in insufficient information within each block. Overall, the experimental results indicate that increasing the number of blocks improves the model's ability to capture dynamic changes from different temporal positions. Carefully selecting the window size and stride enables the model to balance information richness and computational efficiency, thereby significantly improving decoding performance.

### 4.5 Model efficiency analysis

In the evaluation of deep learning models, performance is comprehensively assessed through multiple metrics such as accuracy, number of parameters, and inference time. The number of parameters refers to the total count of learnable parameters in the model, mainly including the weights and biases of the neural network layers. When sufficient data are available, models with a larger number of parameters possess stronger fitting and feature representation capabilities, enabling them to capture subtle data patterns and improve accuracy. However, excessive parameters consume more memory, require higher hardware capacity, and increase computational load during inference, thereby reducing efficiency. Inference time refers to the duration required for a model to generate an output after receiving an input, measured from the completion of forward propagation. It is influenced by multiple factors such as computational complexity, hardware performance, and software optimization. Inference time directly reflects the model's data processing speed and serves as a key indicator of computational efficiency. In this paper, the inference time of all models refers to the batch-level average value (in milliseconds). Specifically, the measurement procedure involves feeding batches of trial-segment data into the model and recording the total elapsed time from data input to output generation.

**Figure 13:**
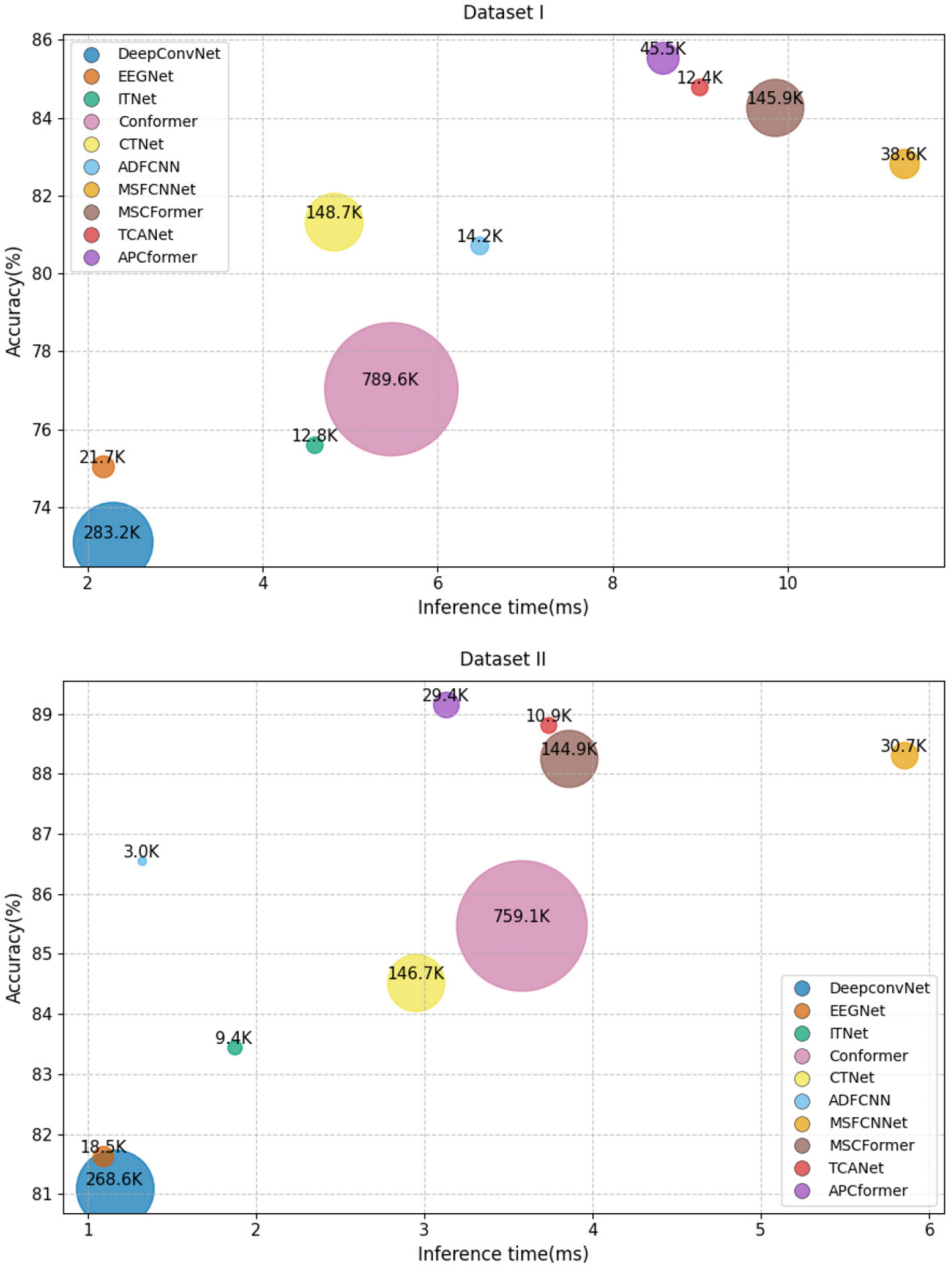
Comparison of classification accuracy, parameter quantity and inference time of APCformer. Among them, the vertical coordinate represents the accuracy rate, the horizontal coordinate represents the inference time, the bubble size represents the parameter quantity of the model, and the K in the bubble represents 1 × 10^3^.

Figure 13 presents a comparison of APCformer's accuracy, parameter count, and inference time on Dataset I and Dataset II. Specifically, in Dataset I, APCformer has a parameter count of 45.5K, which is significantly lower than that of Conformer (789.6K), DeepConvNet (283.2K), CTNet (148.7K), and MSCFormer (145.9K). Its inference time is 8.57ms, slightly higher than ADFCNN (6.48ms), Conformer (5.46ms), and CTNet (4.81ms). In Dataset II, APCformer's parameter count (29.4K) is higher only than ADFCNN (3.0K), ITNet (9.4K), TCANet (10.9K), and EEGNet (18.5K), while its inference time (3.13ms) is lower than that of Conformer (3.58ms), MSCFormer (3.86ms), TCANet (3.74ms), and MSFCNNet (5.85ms). It can be clearly observed that APCformer achieves the highest accuracy among all baseline methods while maintaining a relatively low parameter count and moderate inference time, demonstrating a favorable balance between model complexity and performance. Compared with Conformer, APCformer has significantly fewer parameters, showing a 94.25% reduction on Dataset I, while maintaining a similar inference time and achieving an accuracy improvement of 8.47%. On Dataset II, the parameter count decreases by 96.13%, and the inference time is reduced by 12.57%. When compared with MSFCNNet, APCformer shows a 17.72% increase in parameter count on Dataset I, but the inference time is shortened by 24.36%. On Dataset II, the parameter counts of the two models are nearly the same, with a difference of only 1.35K, while APCformer achieves a 46.49% reduction in inference time. Compared with MSCFormer, APCformer has significantly reduced the number of parameters on both datasets, and the inference time has also been improved to a certain extent. Although APCformer ranks in the middle in terms of overall inference time, it outperforms MSFCNNet, MSCFormer and TCANet in both inference time and accuracy. This performance gain primarily results from the integration of multiple architectural components, such as the attention mechanism and multi-scale feature interaction fusion. These modules enhance the model's capability for core feature extraction and contribute to higher accuracy, though they inevitably increase computational complexity. Overall, APCformer achieved high accuracy on both datasets, and the number of parameters and inference time did not increase excessively. It has achieved a good balance among accuracy, the number of parameters, and inference time.

### 4.6 Model visual analysis

**Figure 14:**
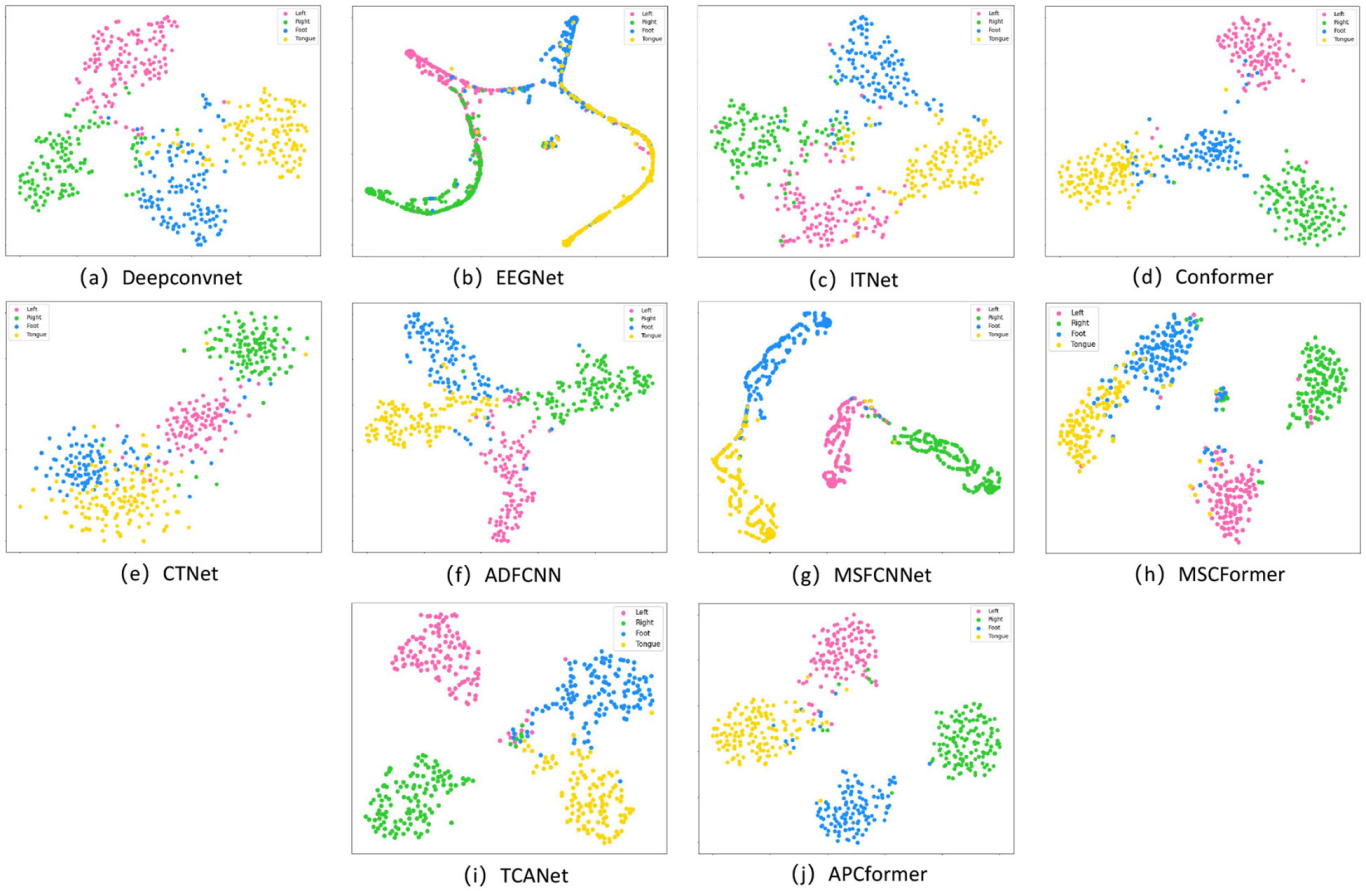
The t-SNE visualizes the distribution of model features. **(a)** DeepConvNet, **(b)** EEGNet, **(c)** ITNet, **(d)** Conformer, **(e)** CTNet, **(f)** ADFCNN, **(g)** MSFCNNet, **(h)** MSCFormer, **(i)** TCANet, and **(j)** APCformer.

To visually reveal the separability of features by different models, this paper employs the t-SNE (Van der Maaten and Hinton, 2008) dimensionality reduction and visualization technique to map the high-dimensional features output by each model into a two-dimensional space. The degree of separation between classes and the compactness within classes are analyzed. The visualization results are shown in Figure 14. The feature distributions of the baseline models generally suffer from insufficient category discrimination, which is largely related to their structural design. In pure CNN models, DeepConvNet and EEGNet have fixed receptive fields and can only capture very limited local features. As shown in Figures 14a, b, the features of the same category are scattered, and features of different categories are intertwined in large quantities. Although ITNet uses Inception modules to expand the convolutional perception range, it is still limited to extracting local features and ignores the temporal correlation of EEG signals. As shown in Figure 14c, the feature clusters are loosely aggregated, and there are still many overlaps between classes, revealing the insufficiency of pure CNN networks in global correlation modeling. In the CNN-Transformer-based models, although Conformer and CTNet recognize the importance of global features of EEG signals, they still use fixed convolution to extract local features. As shown in Figures 14d, e, both exhibit blurred class boundaries and scattered features of the same category, indicating that simply fusing CNN and Transformer cannot effectively optimize local and global features in a coordinated manner.

On this basis, the multi-scale fusion models ADFCNN, MSFCNNet, MSCFormer, and TCANet expand the feature dimensions in a cascaded manner, solving the problems of local feature extraction and global feature correlation, but they lack important feature selection and cross-scale interaction mechanisms, resulting in the masking of fine-grained features. As shown in Figures 14f–i, the results show insufficient intra-class compactness and indistinct inter-class distances, with some feature clusters showing local overlapping. In contrast to the baseline models, APCformer is capable of effectively addressing these shortcomings. As shown in Figure 14j, the results present an ideal state of strong inter-class separation and high intra-class compactness, with sharp and distinguishable class boundaries and low dispersion, further confirming the superiority and robustness of APCformer.

## 5 Discussion

In this paper, we propose an Aggregation-Perception Enhanced Convolutional Transformer network for MI-EEG signal decoding (APCformer). The model effectively addresses the limitations of traditional CNNs, such as the lack of cross-scale interaction, insufficient capture of long-range dependencies, and the tendency to lose fine-grained local features. At the same time, it improves upon Transformer-based approaches by enhancing temporal dependency modeling for EEG signals and achieving a better balance between computational efficiency and performance. The proposed method adopts a multi-scale feature interaction and fusion architecture that progressively refines representations in a hierarchical manner. The STConv and AFR modules enable the network to capture coarse-grained features, while the PAE module further refines these features and injects positional encoding information to strengthen temporal information processing. The SAT module integrates Aggregation-Attention and Top-Attention mechanisms to enhance long-range dependencies and local correlations among features, while maintaining computational efficiency. Together, these components comprehensively improve both the depth and breadth of EEG feature analysis. This design enables APCformer to achieve end-to-end network performance that surpasses the existing mainstream CNN-Transformers.

Experimental results show that APCformer achieves an average accuracy of 85.53% on the BCI-IV 2a dataset and 89.15% on the BCI-IV 2b dataset, significantly outperforming existing mainstream methods. Moreover, the evaluation conducted using the kappa coefficient indicates that APCformer has outstanding performance. Compared with methods that rely solely on CNNs, APCformer effectively compensates for the limitations in modeling long-range dependencies by incorporating a Transformer architecture. Furthermore, in contrast to conventional CNN–Transformer hybrid approaches, APCformer achieves more refined feature extraction and optimization through its multi-scale feature fusion and sparse information aggregation mechanisms.

The results of the baseline comparison experiments show that CNN-based methods such as DeepConvNet, EEGNet, and ITNet, which fail to adequately capture the global dependencies of EEG signals, exhibit limited decoding accuracy of 74.22%, 76.39%, and 77.66%, respectively. In contrast, models integrating Transformer architectures, such as Conformer and CTNet, show better temporal modeling performance, achieving accuracies of 78.57% and 81.92%, though they still lack sufficient attention to fine-grained feature representations. Multi-scale CNN–Transformer hybrid models, including ADFCNN, MSFCNNet, MSCFormer, and TCANet, further improve accuracy to 81.42%, 83.32%, 84.26%, and 84.78%, benefiting from more flexible feature fusion strategies. However, they still have limitations in establishing effective cross-scale correlations and balancing the relationship between global and local characteristics. By contrast, APCformer, with its distinctive sparse information aggregation mechanism, effectively overcomes these limitations, achieving the highest decoding accuracy while maintaining a relatively low parameter count, demonstrating superior efficiency and representational capability.

The ablation study results demonstrate the significant contribution of each module within the APCformer architecture. The complete APCformer model achieves the best overall performance, with all modules contributing positively. Among them, the SAT module plays the most critical role, its removal leads to a 4.67% drop in accuracy, confirming its importance in handling both global and local feature interactions. The removal of the PAE and AFR modules results in accuracy decreases of 1.68% and 1.52%, respectively, highlighting their essential roles in extracting deep fine-grained features. The use of the interaction mechanism enables the network to effectively prevent the loss of critical information, resulting in an 1.32% increase in accuracy. Notably, using only the multi-scale convolution module yields an accuracy of 77.66%, which still surpasses that of the single-scale EEGNet, further validating the effectiveness of the multi-scale architectural design in improving EEG feature representation. Moreover, the confusion matrix results show that APCformer achieves high recognition accuracy across all classes, with a particularly notable improvement after integrating the SAT module, validating its effectiveness in enhancing the spatial discriminability of EEG features.

The parameter sensitivity analysis provides important insights for guiding the practical application of APCformer. In the analysis of frequency bands and channel numbers, the experiments reveal that model accuracy increases with the number of EEG channels. Appropriately integrating information from more channels and wider frequency bands can effectively enhance model performance. In the comparison of convolution kernel sizes, the multi-scale parallel structure demonstrates superior performance and shows a clear advantage over single-scale configurations, confirming the importance of proper kernel-scale selection in improving model accuracy. The hyperparameter analysis of the SAT module indicates that the number of attention layers and heads has a relatively minor effect on model performance. However, an excessive number of layers or heads significantly increases computational complexity. These findings provide valuable reference points for practical hyperparameter selection and model optimization in EEG decoding applications.

The efficiency analysis results indicate that APCformer achieves a well-balanced trade-off among accuracy, parameter count, and inference time. Across both datasets, the model maintains parameter counts of 45.5K and 29.4K, respectively, while keeping inference time at a moderate level. This efficiency advantage makes APCformer more feasible in practical BCI applications. Moreover, the t-SNE visualization further illustrates that the features extracted by APCformer exhibit clearer class clustering and higher separability, providing additional evidence of the model's superior feature extraction and representation capabilities.

This study still has several limitations. First, the research relies on subject-specific task scenarios, requiring separate training for each individual and not leveraging shared EEG feature patterns across multiple subjects. Due to the limited scale of the used EEG dataset, the deep learning model lacks sufficient data iteration, fails to fully verify the model's cross-session generalization ability, has a certain risk of information leakage, and may overestimate the model's performance in practical deployment. In the future, we will explore its cross-session and cross-subject applications, and introduce a transfer learning framework to pre-train a general feature extractor, so as to further enhance the decoding performance of the model. Second, the model contains numerous and sensitive hyperparameters, such as the multi-scale convolution kernel combinations in the PAE module and the sliding window parameters in the SAT module, which require extensive manual tuning and are both time- and computation-intensive. Future research will focus on optimizing the core components and employing automated hyperparameter optimization to shorten the tuning cycle and improve efficiency. Third, the model's inference efficiency and lightweight adaptability remain limited. Although APCformer achieves high decoding accuracy, its parameter count and inference time are less competitive than those of lightweight models. While the current performance meets the basic requirements for BCI tasks, there is still substantial room for improvement in real-world applications. Future work will focus on lightweight model redesign by simplifying structural complexity and reducing parameter redundancy to improve adaptability and robustness for deployment on wearable and embedded BCI devices.

## 6 Conclusion

This paper proposes a novel APCformer network model for EEG signal decoding, which demonstrates excellent performance in EEG decoding tasks. APCformer jointly extracts the spatiotemporal dynamic features of EEG signals through multi-scale convolution and attention modules. Using parallel convolutions, it captures the deep characteristics of EEG signals, while positional encoding enhances feature associations. The SAT module is employed for sequence modeling to strengthen temporal information processing. All components work together to achieve progressive optimization from global coarse-grained to local fine-grained feature representations. Comparative experiments conducted on two public datasets show that APCformer improves both the efficiency and accuracy of EEG decoding, demonstrating strong learning capability in EEG decoding tasks. Ablation experiments further confirm the positive contribution of each module, validating their necessity. Although APCformer does not achieve the smallest parameter count or the shortest inference time among all compared methods, it maintains an excellent balance between accuracy and computational complexity. Combined with visualization analysis, the results further verify the effectiveness of the proposed method. In summary, APCformer demonstrates outstanding performance in EEG signal decoding tasks and shows great potential as a new candidate for BCI applications.

## Data Availability

The original contributions presented in the study are included in the article/supplementary material, further inquiries can be directed to the corresponding author.

## References

[B1] Altaheri H. Muhammad G. Alsulaiman M. (2022). Physics-informed attention temporal convolutional network for EEG-based motor imagery classification. IEEE Trans. Ind. Inform. 19, 2249–2258. doi: 10.1109/TII.2022.3197419

[B2] Beltagy I. Peters M. E. Cohan A. (2020). Longformer: The long-document transformer. arXiv preprint arXiv:2004.05150. doi: 10.48550/arXiv.2004.05150

[B3] Brunner C. Leeb R. Müller-Putz G. Schlögl A. Pfurtscheller G. (2008). BCI competition 2008-graz data set a. Instit. Knowl. Disc. Graz Univ. Technol. 16:34. doi: 10.21227/katb-zv89

[B4] Cai X. Ma S. Cao L. Li J. Liu T. Dong Y. (2025). Fusion of multiscale features via centralized sparse-attention network for EEG decoding. arXiv preprint arXiv:2512.18689. doi: 10.48550/arXiv.2512.18689

[B5] Chen J. Yu Z. Gu Z. Li Y. (2020). Deep temporal-spatial feature learning for motor imagery-based brain-computer interfaces. IEEE Trans. Neural Syst. Rehabilit. Eng. 28, 2356–2366. doi: 10.1109/TNSRE.2020.3023417 32956061

[B6] Chen K. Chai S. Cai M. Liu Q. Ai Q. Zhou C. . (2025). A novel 3D feature fusion network for EEG emotion recognition. Biomed. Signal Process. Control 102:107347. doi: 10.1016/j.bspc.2024.107347

[B7] Chen X. Li H. Li M. Pan J. (2023). “Learning a sparse transformer network for effective image deraining,” in 2023 IEEE/CVF Conference on Computer Vision and Pattern Recognition (CVPR), 5896–5905. doi: 10.1109/CVPR52729.2023.00571

[B8] Dosovitskiy A. Beyer L. Kolesnikov A. Weissenborn D. Zhai X. Unterthiner T. . (2020). An image is worth 16x16 words: transformers for image recognition at scale. arXiv preprint arXiv:2010.11929.

[B9] Geng X. Li D. Chen H. Yu P. Yan H. Yue M. (2022). An improved feature extraction algorithms of EEG signals based on motor imagery brain-computer interface. Alexandr. Eng. J. 61, 4807–4820. doi: 10.1016/j.aej.2021.10.034

[B10] Gong L. Li M. Zhang T. Chen W. (2023). EEG emotion recognition using attention-based convolutional transformer neural network. Biomed. Signal Process. Control 84:104835. doi: 10.1016/j.bspc.2023.104835

[B11] Guan S. Dong T. Cong L.-K. (2025). Method for EEG signal recognition based on multi-domain feature fusion and optimization of multi-kernel extreme learning machine. Sci. Rep. 15:6601. doi: 10.1038/s41598-025-87569-5 39994209 PMC11850847

[B12] Guo S. Wang Y. Zhang X. Tang B. (2025). A cross-session non-stationary attention-based motor imagery classification method with critic-free domain adaptation. Biomed. Signal Process. Control 100:107122. doi: 10.1016/j.bspc.2024.107122

[B13] Han C. Liu C. Wang J. Wang Y. Cai C. Qian D. (2025). A spatial-spectral and temporal dual prototype network for motor imagery brain-computer interface. Knowl. Based Syst. 315:113315. doi: 10.1016/j.knosys.2025.113315

[B14] Herman P. Prasad G. McGinnity T. M. Coyle D. (2008). Comparative analysis of spectral approaches to feature extraction for EEG-based motor imagery classification. IEEE Trans. Neural Syst. Rehabilit. Eng. 16, 317–326. doi: 10.1109/TNSRE.2008.926694 18701380

[B15] Hsu W.-Y. Cheng Y.-W. (2023). EEG-channel-temporal-spectral-attention correlation for motor imagery EEG classification. IEEE Trans. Neural Syst. Rehabilit. Eng. 31, 1659–1669. doi: 10.1109/TNSRE.2023.3255233 37028310

[B16] Huang W. Deng Y. Hui S. Wu Y. Zhou S. Wang J. (2024). Sparse self-attention transformer for image inpainting. Pattern Recognit. 145:109897. doi: 10.1016/j.patcog.2023.109897

[B17] Ke S. Yang B. Qin Y. Rong F. Zhang J. Zheng Y. (2024). Fact-net: a frequency adapter CNN with temporal-periodicity inception for fast and accurate mi-EEG decoding. IEEE Trans. Neural Syst. Rehabilit. Eng. 32, 4131–4142. doi: 10.1109/TNSRE.2024.3499998 40030248

[B18] Lawhern V. J. Solon A. J. Waytowich N. R. Gordon S. M. Hung C. P. Lance B. J. (2018). EEGnet: a compact convolutional neural network for EEG-based brain-computer interfaces. J. Neural Eng. 15:056013. doi: 10.1088/1741-2552/aace8c 29932424

[B19] Leeb R. Brunner C. Müller-Putz G. Schlögl A. Pfurtscheller G. (2008). BCI competition 2008-graz data set B. Graz Univ. Technol. Austr. 16, 1–6. Available online at: https://api.semanticscholar.org/CorpusID:16768813

[B20] Liu K. Yang T. Yu Z. Yi W. Yu H. Wang G. . (2024). MSVTNet: multi-scale vision transformer neural network for EEG-based motor imagery decoding. IEEE J. Biomed. Health Inform. 28, 7126–7137. doi: 10.1109/JBHI.2024.3450753 39190517

[B21] Liu X.-Y. Wang W.-L. Liu M. Chen M.-Y. Pereira T. Doda D. Y. . (2025). Recent applications of EEG-based brain-computer-interface in the medical field. Milit. Med. Res. 12:14. doi: 10.1186/s40779-025-00598-z 40128831 PMC11931852

[B22] Mansour S. Giles J. Nair K. P. Marshall R. Ali A. Arvaneh M. (2025). A clinical trial evaluating feasibility and acceptability of a brain-computer interface for telerehabilitation in patients with stroke. J. Neuroeng. Rehabil. 22:91. doi: 10.1186/s12984-025-01607-x 40269846 PMC12020174

[B23] Richhariya B. Tanveer M. (2018). EEG signal classification using universum support vector machine. Expert Syst. Appl. 106, 169–182. doi: 10.1016/j.eswa.2018.03.053

[B24] Salami A. Andreu-Perez J. Gillmeister H. (2022). EEG-itnet: an explainable inception temporal convolutional network for motor imagery classification. IEEE Access 10, 36672–36685. doi: 10.1109/ACCESS.2022.3161489

[B25] Schirrmeister R. T. Springenberg J. T. Fiederer L. D. J. Glasstetter M. Eggensperger K. Tangermann M. . (2017). Deep learning with convolutional neural networks for EEG decoding and visualization. Hum. Brain Mapp. 38, 5391–5420. doi: 10.1002/hbm.23730 28782865 PMC5655781

[B26] Song Y. Zheng Q. Liu B. Gao X. (2022). EEG conformer: convolutional transformer for EEG decoding and visualization. IEEE Trans. Neural Syst. Rehabilit. Eng. 31, 710–719. doi: 10.1109/TNSRE.2022.3230250 37015413

[B27] Tang Y. Wang Y. Zhang X. Wang Z. (2023). Stiln: a novel spatial-temporal information learning network for EEG-based emotion recognition. Biomed. Signal Process. Control 85:104999. doi: 10.1016/j.bspc.2023.104999

[B28] Tao W. Wang Z. Wong C. M. Jia Z. Li C. Chen X. . (2023). ADFCNN: attention-based dual-scale fusion convolutional neural network for motor imagery brain-computer interface. IEEE Trans. Neural Syst. Rehabilit. Eng. 32, 154–165. doi: 10.1109/TNSRE.2023.3342331 38090841

[B29] Van der Maaten L. Hinton G. (2008). Visualizing data using t-SNE. J. Mach. Learn. Res. 9, 2579–2605. Available online at: http://jmlr.org/papers/v9/vandermaaten08a.html

[B30] Vaswani A. Shazeer N. Parmar N. Uszkoreit J. Jones L. Gomez A. N. . (2017). “Attention is all you need,” *Advances in Neural Information Processing Systems*, 30. doi: 10.48550/arXiv.1706.03762

[B31] Wei X. Dong E. Zhu L. (2021). “Multi-class MI-EEG classification: using fbcsp and ensemble learning based on majority voting,” in 2021 China Automation Congress (CAC), 872–876. doi: 10.1109/CAC53003.2021.9728576

[B32] Wilcoxon F. (1992). “Individual comparisons by ranking methods,” in Breakthroughs in Statistics: Methodology and Distribution (New York, NY: Springer New York), 196–202. doi: 10.1007/978-1-4612-4380-9_16

[B33] Willett F. R. Avansino D. T. Hochberg L. R. Henderson J. M. Shenoy K. V. (2021). High-performance brain-to-text communication via handwriting. Nature 593, 249–254. doi: 10.1038/s41586-021-03506-2 33981047 PMC8163299

[B34] Woo S. Park J. Lee J.-Y. Kweon I. S. (2018). “CBAM: convolutional block attention module,” in Proceedings of the European Conference on Computer Vision (ECCV), 3–19. doi: 10.1007/978-3-030-01234-2_1

[B35] Xie J. Zhang J. Sun J. Ma Z. Qin L. Li G. . (2022). “A transformer-based approach combining deep learning network and spatial-temporal information for raw EEG classification. IEEE Trans. Neural Syst. Rehabilit. Eng. 30, 2126–2136. doi: 10.1109/TNSRE.2022.3194600 35914032

[B36] Yang G. Liu J. (2024). A novel multi-scale fusion convolutional neural network for EEG-based motor imagery classification. Biomed. Signal Process. Control 96:106645. doi: 10.1016/j.bspc.2024.106645

[B37] Zhang Y. Chen W. Lin C.-L. Pei Z. Chen J. Chen Z. (2021). Boosting-lda algriothm with multi-domain feature fusion for motor imagery EEG decoding. Biomed. Signal Process. Control 70:102983. doi: 10.1016/j.bspc.2021.102983

[B38] Zhao D. Tang F. Si B. Feng X. (2019). Learning joint space-time-frequency features for EEG decoding on small labeled data. Neural Netw. 114, 67–77. doi: 10.1016/j.neunet.2019.02.009 30897519

[B39] Zhao P. Lian C. Xu B. Zeng Z. (2024). Multiscale global prompt transformer for EEG-based driver fatigue recognition. IEEE Trans. Autom. Sci. Eng. 22, 2700–2711. doi: 10.1109/TASE.2024.3382892

[B40] Zhao W. Jiang X. Zhang B. Xiao S. Weng S. (2024). Ctnet: a convolutional transformer network for EEG-based motor imagery classification. Sci. Rep. 14:20237. doi: 10.1038/s41598-024-71118-7 39215126 PMC11364810

[B41] Zhao W. Lu H. Zhang B. Zheng X. Wang W. Zhou H. (2025a). Tcanet: a temporal convolutional attention network for motor imagery EEG decoding. Cogn. Neurodyn. 19:91. doi: 10.1007/s11571-025-10275-5 40524963 PMC12167204

[B42] Zhao W. Zhang B. Zhou H. Wei D. Huang C. Lan Q. (2025b). Multi-scale convolutional transformer network for motor imagery brain-computer interface. Sci. Rep. 15:12935. doi: 10.1038/s41598-025-96611-5 40234486 PMC12000594

[B43] Zhao Z. Cao Y. Yu H. Yu H. Huang J. (2025). CNNVIT-MILF-a: a novel architecture leveraging the synergy of CNN and VIT for motor imagery classification. IEEE J. Biomed. Health Inform. 30, 1153–1165. doi: 10.1109/JBHI.2025.3587026 40627473

